# Identification of age- and disease-related alterations in circulating miRNAs in a mouse model of Alzheimer's disease

**DOI:** 10.3389/fncel.2015.00053

**Published:** 2015-02-19

**Authors:** Sylvia Garza-Manero, Clorinda Arias, Federico Bermúdez-Rattoni, Luis Vaca, Angélica Zepeda

**Affiliations:** ^1^Departamento de Medicina Genómica y Toxicología Ambiental, Instituto de Investigaciones Biomédicas, Universidad Nacional Autónoma de MéxicoCoyoacán, México; ^2^División de Neurociencias, Instituto de Fisiología Celular, Universidad Nacional Autónoma de MéxicoCoyoacán, México; ^3^Departamento de Biología Celular, Instituto de Fisiología Celular, Universidad Nacional Autónoma de MéxicoCoyoacán, México

**Keywords:** early diagnosis, neurodegenerative diseases, blood-based biomarker, Alzheimer models, plasma, pathological aging, prognosis, 3x-Tg

## Abstract

Alzheimer's disease (AD) is a neurodegenerative disorder characterized clinically by the progressive decline of memory and cognition. Histopathologically, two main hallmarks have been identified in AD: amyloid-β peptide extracellular neuritic plaques and neurofibrillary tangles formed by posttranslational modified tau protein. A definitive diagnosis can only be achieved after the *post mortem* verification of the histological mentioned alterations. Therefore, the development of biomarkers that allow an early diagnosis and/or predict disease progression is imperative. The prospect of a blood-based biomarker is possible with the finding of circulating microRNAs (miRNAs), a class of small non-coding RNAs of 22–25 nucleotides length that regulate mRNA translation rate. miRNAs travel through blood and recent studies performed in potential AD cases suggest the possibility of finding pathology-associated differences in circulating miRNA levels that may serve to assist in early diagnosis of the disease. However, these studies analyzed samples at a single time-point, limiting the use of miRNAs as biomarkers in AD progression. In this study we evaluated miRNA levels in plasma samples at different time-points of the evolution of an AD-like pathology in a transgenic mouse model of the disease (3xTg-AD). We performed multiplex qRT-PCR and compared the plasmatic levels of 84 miRNAs previously associated to central nervous system development and disease. No significant differences were detected between WT and transgenic young mice. However, age-related significant changes in miRNA abundance were observed for both WT and transgenic mice, and some of these were specific for the 3xTg-AD. In agreement, variations in the levels of particular miRNAs were identified between WT and transgenic old mice thus suggesting that the age-dependent evolution of the AD-like pathology, rather than the presence and expression of the transgenes, modifies the circulating miRNA levels in the 3xTg-AD mice.

## Introduction

Alzheimer's disease (AD) is the most common cause of dementia clinically characterized by the progressive decline of memory. At the histopathological level two main hallmarks define it: the abnormal extracellular accumulation of amyloid-β peptide (Aβ) into neuritic plaques and the formation of intraneuronal neurofibrillary tangles (NFTs) composed by posttranslational modified tau protein. These pathological markers are mainly located in hippocampus and cortical regions (Selkoe, 2001; Ballard et al., 2011) and are accompanied by synaptic loss early in the disease, which constitutes the major morphological correlate of memory decline (Terry et al., 1991; Dekosky et al., 1996). AD is the major neurodegenerative disorder in the elderly; the prevalence of the disease increases with age, thus aging constitutes the main risk factor (World Alzheimer Report, ADI, 2009). Two recognized forms of the disease include the sporadic AD (SAD) and the familial AD (FAD). SAD accounts for more than 99% of total cases and is characterized by a late-onset of symptomatology, whereas FAD is an early-onset form of the disease associated with mutations in three genes encoding for the amyloid precursor protein (APP), presenilin 1 (PS1), and presenilin 2 (PS2) transmitted in an autosomal dominant pattern (Goate et al., 1991; Levy-Lahad et al., 1995; Rogaev et al., 1995; Sherrington et al., 1995).

Currently, amyloid-imaging positron emission tomography (PET) through the Pittsburgh Compound-B (PIB) tracer is the best tool for identifying amyloid deposits in patients (Klunk et al., 2004; Alkalay et al., 2013). However, a definitive diagnosis of AD can only be achieved after *post mortem* histopathological verification. Therefore, the development of sensitive and specific non-invasive methods to detect molecular markers or to best predict the disease progression is crucial. Among different prospects of a blood-based biomarker, the finding that circulating microRNAs (miRNAs) derived from different types of cells can be detected, make possible their use as non-invasive markers of brain dysfunction. miRNAs are small non-coding RNAs of 22–25 nucleotides length that posttrancriptionally regulate the level of many proteins via mRNA silencing or degradation (Bartel, 2009; Fabian and Sonenberg, 2012; Ameres and Zamore, 2013). They travel through blood within specialized vesicles known as exosomes, associated with protein complexes or into high density lipoproteins as well (Valadi et al., 2007; Arroyo et al., 2011; Vickers et al., 2011). Because endogenous miRNAs are protected in circulation, they are highly stable and their levels can be analyzed by conventional techniques of molecular biology through which changes in circulating miRNA levels have been associated with diverse pathologies (Chen et al., 2008; Lawrie et al., 2008; Mitchell et al., 2008).

Analysis of miRNA profiles in *post mortem* brain and cerebrospinal fluid (CSF) samples of AD patients have revealed changes in miRNA levels associated to the disease (Lukiw, 2007; Cogswell et al., 2008; Hébert et al., 2008; Wang et al., 2008b, 2011; Nunez-Iglesias et al., 2010; Alexandrov et al., 2012; Müller et al., 2014). Recent studies performed in plasma samples of potential AD cases suggest the possibility of finding differences between circulating miRNA levels of AD cases and control subjects (Geekiyanage et al., 2011; Kumar et al., 2013; Leidinger et al., 2013; Tan et al., 2013; Burgos et al., 2014; Kiko et al., 2014). In this regard, studies have aimed at analyzing miRNA levels at a single time-point, which may limit their use as biomarkers or as disease predictors. The possibility to evaluate circulating miRNA levels at different time-points in the progression of the disease could provide information regarding key physiopathological features of AD and could contribute in establishing early blood-based molecular biomarkers for the disease. Analyzing miRNA levels in blood samples from patients at an early time-point is hampered by the fact that symptoms do not develop until the disease is at an advanced stage. Thus, in this study we used the triple transgenic mouse model (3xTg-AD) which reproduces the age-dependent progress of amyloid pathology and develops the tauopathy similar to that found in AD (Oddo et al., 2003). In order to evaluate specific miRNA levels in plasma samples at different time-points of the evolution of the AD-like pathology, we performed multiplex qRT-PCR and compared the levels from 3xTg-AD and WT mice of 84 miRNAs previously associated to central nervous system (CNS) development (Giraldez et al., 2005; Yoo et al., 2009; Zhao et al., 2009; Edbauer et al., 2010) and diseases such as schizophrenia (Beveridge et al., 2010), Huntington disease (Johnson et al., 2008), Parkinson disease (Kim et al., 2007; Wang et al., 2008a), and AD.

No significant differences in circulating miRNA levels were detected between 3xTg-AD and WT young mice. However, age-related significant changes in the abundance of certain plasmatic miRNAs were observed in both WT and transgenic mice. Interestingly, some of the miRNAs that changed were specific for the 3xTg-AD. In agreement, variations in the abundance of particular miRNAs were identified between WT and transgenic old mice thus suggesting that the age-dependent evolution of AD-like pathology, rather than the presence and expression of human transgenes, modifies the circulating miRNA profile in the 3xTg-AD mice.

## Materials and methods

### Animals

Homozygous 3xTg-AD developed by Oddo et al. (2003) (*n* = 14) and wild-type (WT) (*n* = 13) male mice (strain 129/C57BL6) were used in the study. For developing the transgenic mice, human APP cDNA harboring the Swedish double mutation (KM670/671NL) and human tau cDNA harboring the P301L mutation were cloned into Thy1.2 expression cassette and co-microinjected into single-cells embryos harvested from homozygous PS1*_M146V_* knockin (KI) mice generated as a hybrid 129/C57BL6 background. For this study, all used 3xTg-AD mice were genotyped by identifying the three harbored human transgenes (Supplementary Figure 1). Brains from four 3xTg-AD male mice 2–3 (*n* = 2) and 14–15 (*n* = 2) months old were used for immunohistochemical analysis. Determination of circulating miRNA profiles included blood samples from six-seven animals per group and a total of four experimental groups were integrated: 2–3 months old-3xTg-AD mice (*n* = 6), 2–3 months old-WT mice (*n* = 6), 14–15 months old-3xTg-AD (*n* = 6), and 14–15 months old-WT mice (*n* = 7). The experiments were performed in accordance with local government rules and the Society for Neuroscience Guide for the Care and Use of Laboratory Animals with approval of the Animal Care Committee of the Instituto de Investigaciones Biomedicas, UNAM. Efforts were made to minimize animal suffering and to reduce the number of subjects used.

### Genotyping

Genomic DNA was isolated from the 3xTg-AD mice tails (2–3 mm) by the *Hot-Shot* method. Tail samples were homogenized with alkaline lysis reagent (25 mM NaOH, 0.2 mM EDTA, pH 12) through mechanical action and incubated during 1 h at 95°C. Tris-HCl buffer (40 mM, pH 5) was added to each sample for neutralization (pH 7.5). Samples were centrifuged during 2 min at 12 500 rpm, 4°C, and the supernatant was used for PCR amplification. Human APP_Swe_ and tau_P301L_ transgenes were identified through their amplification products of 500 bp and 320 bp, respectively into a 2% agarose gel stained with ethidium bromide. The primers used for APP-tau PCR were 5tauRev (5′-TCCAAAGTTCACCTGATAGT-3′), APPinternal (5′-GCTTGCACCAGTTCTGGATGG-3′) and Thy12.4 (5′-GAGGTATTCAGTCATGTGCT-3′). PS1_M146V_ KI was detected by a PCR using the PS1-K13 (5′-CACACGCACACTCTGACATGCACAGGC-3′) and PS1-K15 (5′-AGGCAGGAAGATCACGTGTTCCAAGTAC-3′) primers, followed by an enzymatic digestion (1 h, 60°C) with endonuclease BstEII (New England Biolabs). Products were separated into a 2% agarose gel, and identified as a 530 bp band from WT PS1, or 350 bp and 180 bp bands from mutation carriers PS1.

### Immunohistochemistry

3xTg-AD mice (*n* = 4) were anesthetized with sodium pentobarbital and transcardially perfused with 0.9% chilled saline followed by 4% chilled formaldehyde in 0.1 M phosphate buffer (pH 7.4). Brains were extracted and post-fixed during 24 h at 4°C. Subsequently, brains were transferred to 15 and 30% sucrose solutions in 0.1 M phosphate buffer for 24 h in each concentration. 30 μm-thick coronal sections were obtained in a cryostat (Microm HM550, Thermo Scientific). Free-floating sections were collected in 24-well plates (Corning) and were washed for 10, 15, and 30 min with 0.15 M PBS and 0.4% PBS-triton solutions. They were then incubated in blocking solution (0.4% PBS-triton, 3% NGS) during 2 h at room temperature, followed by an overnight incubation at 4°C with 1:500 dilution of the primary antibody, either the MAB1560 6E10 clone (Chemicon) which recognizes aminoacids 1–17 from human Aβ peptide, or the MN1020 α-p-PHF-tau AT8 clone (Pierce, Thermo Scientific) which recognizes phosphorylated serine 202 and threonine 205 from human tau protein. Sections were washed as previously detailed and were then incubated in a 1:250 dilution of the secondary α-mouse antibody, either Alexa-488 or Alexa-555 for 2 h at room temperature. Finally, sections were washed for 10, 15, and 30 min in 0.15 M PBS, incubated for 3 min in 30 nM DAPI, and washed again. Immunofluorescence was detected using a Zeiss epifluorescence microscope under 10X and 60X.

### Determination of circulating miRNA levels

#### Plasma collection

Blood samples were collected from 3xTg-AD and WT mice at 2–3 and 14–15 months of age. ~500 μL of blood were obtained from the facial vein of each mouse and collected into a new and sterile 1.5 mL centrifuge tube containing 10 μL of 300 mM EDTA used as anticoagulant. Plasma was separated through two centrifugations, one at low (3000 rpm) and another at high (13,000 rpm) speed, both at 4°C for 10 min. For determining circulating miRNA profiles, equal volumes of plasma samples from two mice within the same experimental group were pooled after plasma separation and a total of three different pools per experimental group were included in the analysis. Pooled samples were stored in ice and immediately treated following the RNA isolation protocol.

#### RNA isolation

RNA was isolated from each pool using the miRNeasy Serum/Plasma Kit (Qiagen) following the manufacturer instructions. Briefly, 200 μL of pooled plasma were homogenized with 1000 μL of Qiazol Lysis Reagent. After homogenization, 3.5 μL of a 1.6 × 10^8^ copies/μL solution of the *C. elegans* miR-39 miRNA mimic used as a spike-in control were added to each sample. Then, a phenol-chloroform extraction was performed by incubating the sample in 200 μL of chloroform for 5 min at room temperature and centrifuging for 15 min at 12,000 rpm at 4°C. The aqueous phase was mixed with 1.5 volumes of 100% ethanol and passed through the RNeasy MinElute spin column. The RNA retained in the membrane was washed with buffer solutions, 80% ethanol and eluted in 14 μL of RNase free water. RNA samples were stored in ice and immediately treated for qRT-PCR protocols.

#### qRT-PCR

miRNA levels from plasma of 3xTg-AD and WT mice 2–3 and 14–15 months old were determined using the miScript miRNA PCR Array System (Qiagen). The System consists in three different kits: the miScript II RT Kit which performs the cDNA synthesis, the miScript SYBR Green PCR Kit which prepares the mix for qPCR reactions, and the Pathway-Focused miScript miRNA PCR Array “Neurological Development and Disease” which detects 84 different miRNAs previously associated with CNS development and disease. For the cDNA synthesis, 1.5 μL of RNA isolated from plasma were used as the starting material of a 20 μL-reaction that consists of a universal synthesis driven by the addition of a poly-A tail and an oligo-dT that selectively retrotranscribes the small RNA molecules (<100 nucleotides) avoiding the retrotranscription of miRNA precursors. The reaction was incubated for 1 h at 37°C followed by a 5 min-incubation at 95°C to inactivate the RTase enzyme. cDNA from each sample was diluted by adding 200 μL of RNase free water and stored at −20°C overnight. After thawing the cDNA on ice, 100 μL of sample were added to the qPCR reaction mix using SYBR Green as a detector. 20 μL of qPCR reaction mix were added to each well of the array. The multiplex qPCR was performed using the Rotor Gene 6000 under the following conditions: pre-incubation for 15 min at 95°C, 40 denaturation-alignment-elongation (15 s at 94°C, 30 s at 55°C, and 30 s at 70°C) cycles and a dissociation melt protocol.

### Data analysis

Data were analyzed using the ΔΔCt (2^−ΔCt^) method of relative quantification with the support of the data analysis software for miScript miRNA PCR Arrays available for users at http://pcrdataanalysis.sabiosciences.com/mirna. The settings were manually fixed at 10 cycles of baseline and 0.02 of threshold line across all PCR runs. The software calculated the ΔCt value between the threshold cycle value (Ct) of each miRNA and the Ct of the *C. elegans* miR-39 miRNA mimic spike-in control used for normalization. The software also calculated the (2^−ΔCt^) value of each miRNA between arrays from different experimental groups. Each experimental group consisted of three samples (*n* = 3) and each sample contained the pooled plasma from two animals. The mean of the relative abundance ± SD of each miRNA contained in the samples (*n* = 3) was calculated. The statistical significance between miRNAs was determined by a *t*-test. A dissociation curve analysis was performed to guarantee the presence of only one PCR product.

## Results

**Figure 1:**
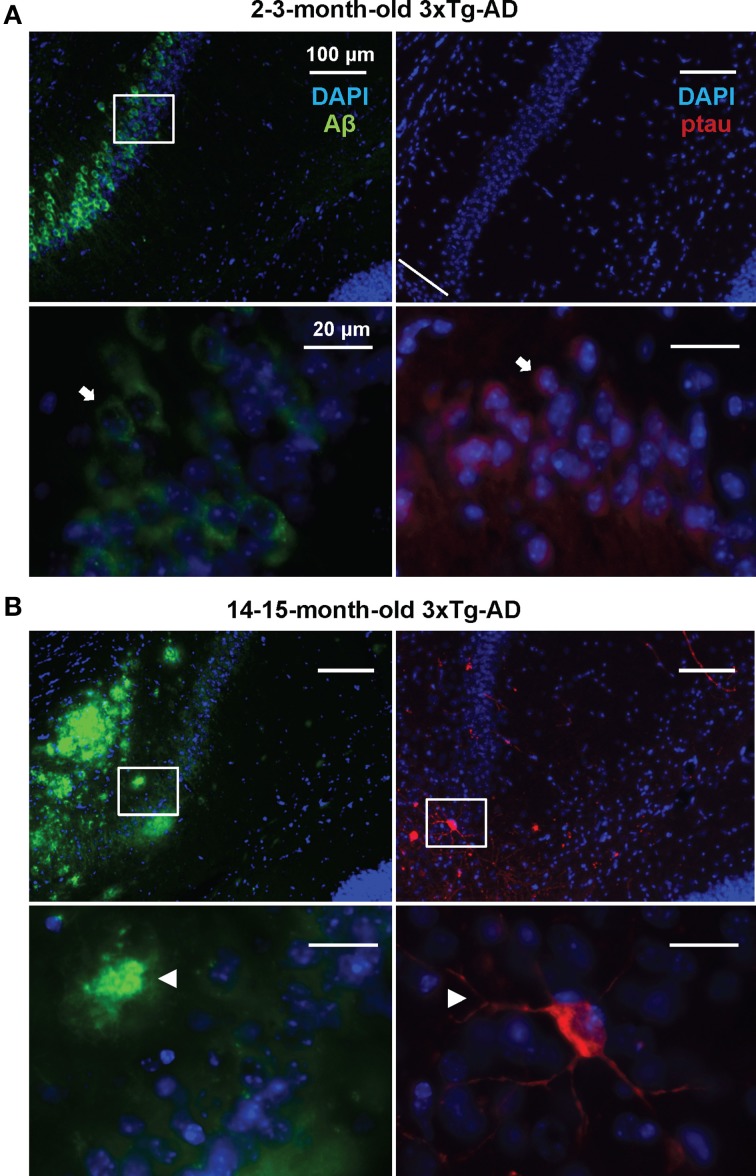
**Development of AD-like histopathological features in the 3xTg-AD**. Brain coronal sections from the hippocampal CA1 field of 3x-Tg-AD mice. **(A)** Two- to three-month-old mice sections show Aβ (green) and p-tau (red) human transgene expression. Bottom panels correspond to magnifications of delineated regions in top images; arrows point at Aβ (left) and p-tau (right) immunostaining with no evidence of AD-like histopathological features. **(B)** Fourteen- to fifteen-month-old mice sections show extracellular aggregates of Aβ (green) and neuronal processes containing p-tau (red). Bottom panels correspond to magnifications of delineated regions in top images; arrowheads point at features indicative of the presence of AD histopathological hallmarks. Scale bars in **(A)** and **(B)**: 100 μm (top images) and 20 μm (bottom images).

In order to detect possible variations in the circulating miRNA profiles related to the AD-like pathology present in the 3xTg-AD transgenic mice, we evaluated the levels of 84 miRNAs previously associated to CNS development and disease in plasma samples of 3xTg-AD and WT mice 2–3 and 14–15 months old. As previously shown (Oddo et al., 2003), these two time-points represent different stages of evolution of the pathological features in the 3xTg-AD mice: at 2–3 months old, histopathological markers are not evidently expressed in the hippocampus whereas at 14–15 months old they become clear in cortical and hippocampal regions. The 2–3 month old-3xTg-AD mice express human transgenes; nevertheless at this time-point we did not find extracellular amyloid aggregates in the hippocampus and scarce phospho-tau (p-tau) immunoreactivity for AT8 was observed (Figure 1A). As previously reported (Oddo et al., 2003) we found that the 14–15 month old-3xTg-AD mice display extracellular amyloid aggregates. Also, neuronal somata and processes were strongly immunopositive for p-tau in AD relevant residues (Figure 1B).

### Abundance of CNS development- and disease-linked miRNAs in mouse plasma

Most of the 84 evaluated miRNAs were detected in mice plasma from all analyzed groups (Supplementary Tables 1–4). Highly abundant detected miRNAs belong to the let-7 family, miR-15 family, miR-30 family, miR-24-27 cluster, miR-29 cluster, miR-17-92 cluster and its paralogs miR-106a-363 and miR-106b-25 that are characterized by their presence in multiple cell lineages as well as by their role in fundamental cellular processes. We found a group of less or not detected miRNAs such as miR-135b, miR-302a/b, miR-488, and miR-9. The plasmatic abundance of detected miRNAs was compared between groups. No significant differences were observed in the circulating miRNA profile from 2–3 months-old 3xTg-AD mice when compared to the age-matched WT group (Supplementary Table 1).

**Figure 2:**
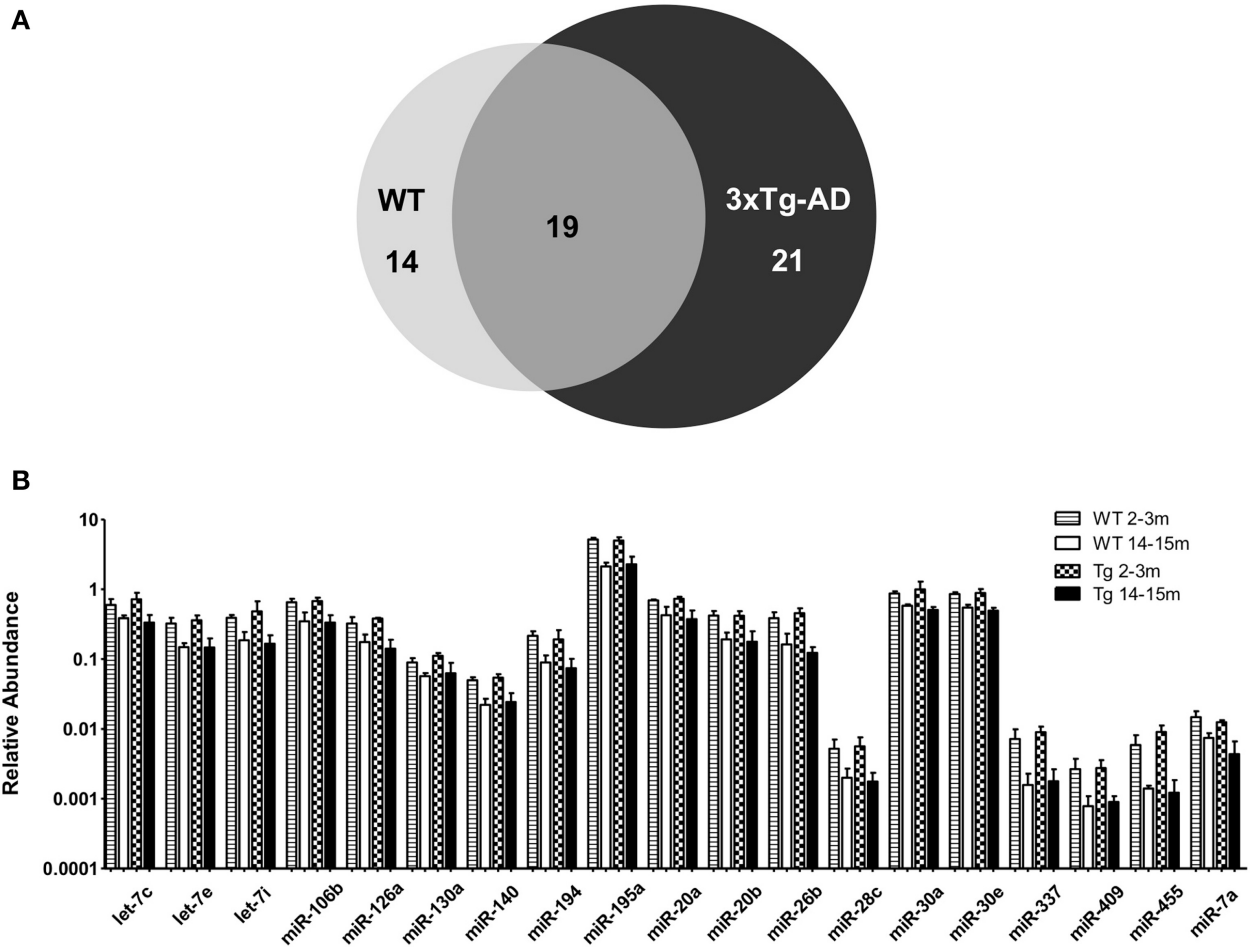
**Significant age-related changes in plasmatic miRNAs in WT and 3x-Tg-AD analyzed by qRT-PCR. (A)** The Venn diagram depicts the number of miRNAs showing statistically different plasma levels between young and old mice in WT (33 miRNAs, in light gray) and in 3xTg-AD mice (40 miRNAs, in black) as shown by a *t*-test; *p* < 0.05. From these miRNAs, 19 are common to both comparisons (dark gray). **(B)** Relative abundance among groups of each of the 19 matching miRNAs. Relative abundance was calculated considering the difference of the Ct of each miRNA and the Ct of the miR-39 of *C. elegans* mimic (ΔCt) using the formula 2^-ΔCt^. miR-39 from *C. elegans* was used as spike-in control in all qRT-PCR experiments. Only miRNAs that showed statistical differences when comparing young and old WT or Tg subjects are shown in the graph; *t*-test; *p* < 0.05.

**Figure 3:**
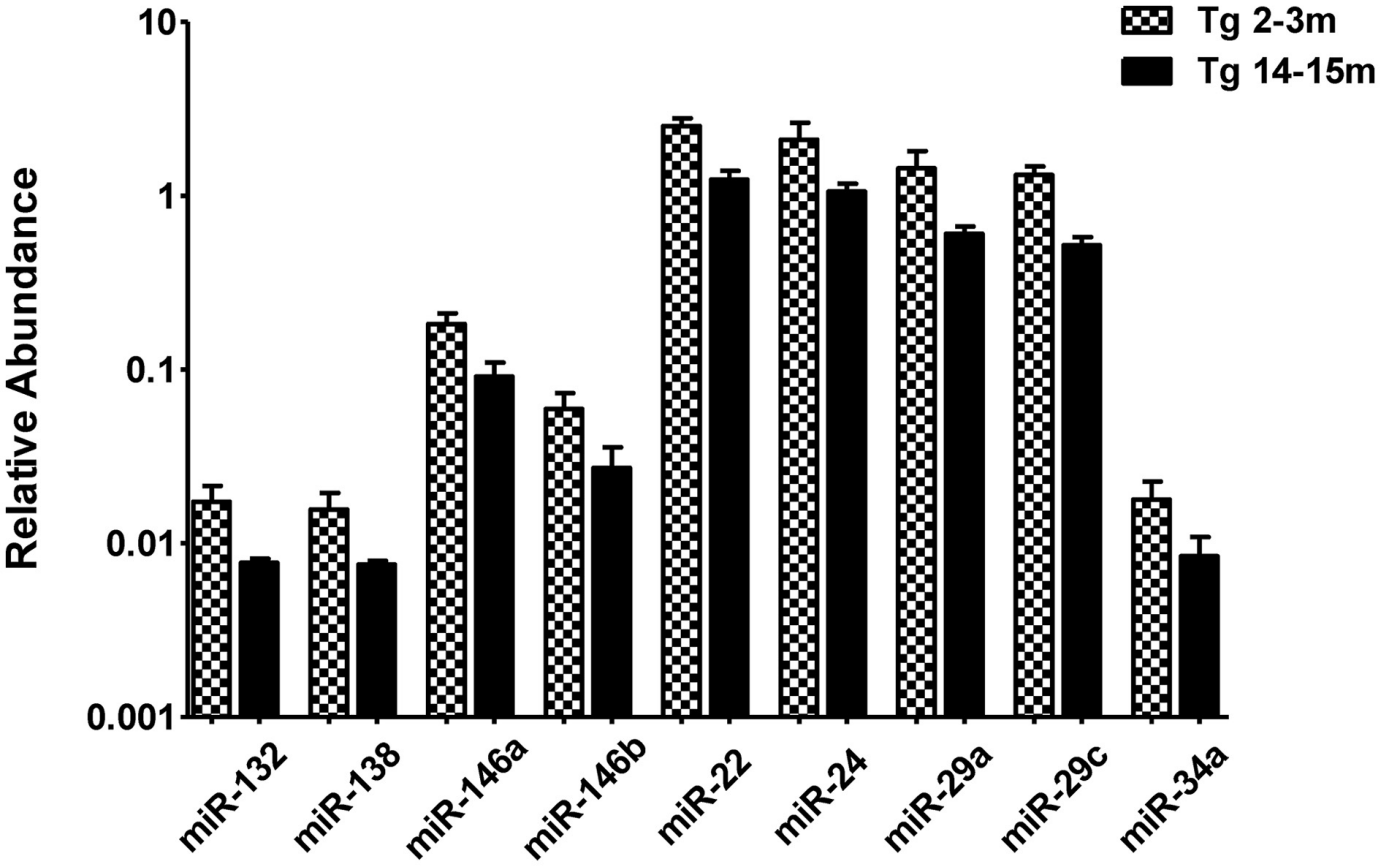
**Significant age-dependent changes in plasmatic miRNAs in the 3xTg-AD analyzed by qRT-PCR**. The graph depicts the relative abundance (mean ± SD) of each one of the miRNAs that show statistically significant differences (*t*-test; *p* < 0.05) in plasma levels between the 2–3 and the 14–15 months-old 3xTg-AD mice. Relative abundances were calculated with the difference of the Ct of each miRNA and the Ct of the miR-39 of *C. elegans* mimics (ΔCt) using the formula 2^-ΔCt^. Determination of circulating miRNA profiles included plasma samples from six-seven animals per group and a total of four experimental groups were integrated: 2–3 months old-3xTg-AD mice (*n* = 3), 2–3 months old-WT mice (*n* = 3), 14–15 months old-3xTg-AD (*n* = 3), and 14–15 months old-WT mice (*n* = 3) (*n* = samples).

When comparing the circulating miRNA profile between the young and the old mice for both WT and 3xTg-AD, we found that aging associated with changes in the plasmatic levels of a group of miRNAs. We detected significant differences in the levels of 33 miRNAs when comparing old vs. young WT mice and in 40 miRNAs when comparing old vs. young 3xTg-AD mice; 19 of these miRNAs were common to both comparisons (Figure 2, Supplementary Tables 2,3). These overlapping miRNAs include family members of let-7, miR-30, miR-17-92 cluster and its paralogs. When comparing young vs. old 3xTg-AD mice, we identified a particular group of miRNAs integrated by miR-132, miR-138, miR-146a, miR-146b, miR-22, miR-24, miR-29a, miR-29c, and miR-34a which show significant differences in plasma levels only in the transgenic group, raising the possibility of age-related changes that specifically occur in the 3xTg-AD mice (Figure 3, Supplementary Table 3).

### Pathology-related changes in the relative abundance of plasmatic miRNAs

**Figure 4:**
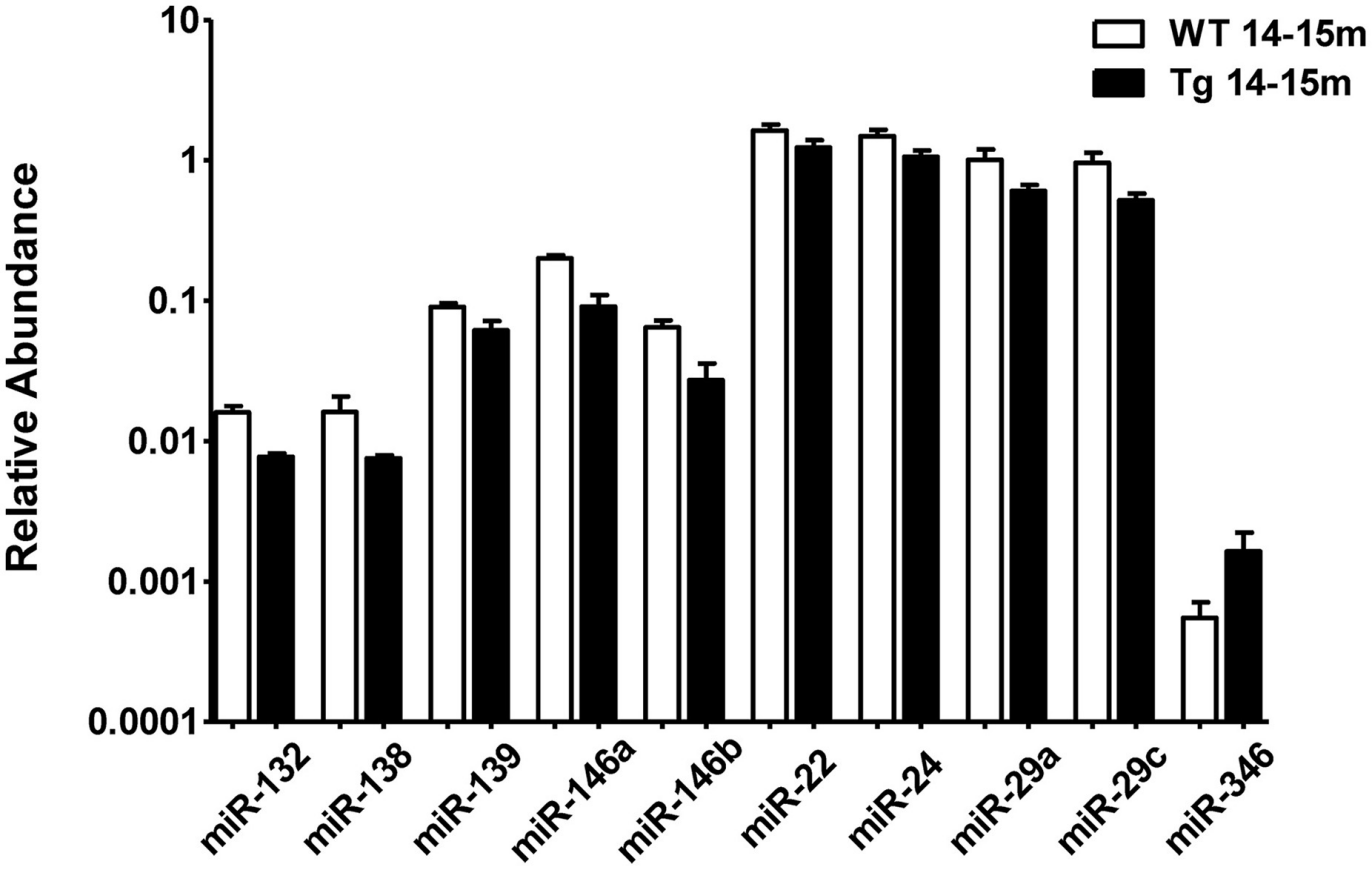
**Significant differences in the abundance of plasmatic miRNAs between aging groups analyzed by qRT-PCR**. The graph depicts the relative abundance (mean ± SD) of each one of the miRNAs that show statistically significant differences (*t*-test; *p* < 0.05) in plasma levels between 14–15 months-old WT and 3xTg-AD mice. Relative abundances were calculated with the difference of the Ct of each miRNA and the Ct of the miR-39 of *C. elegans* mimics (ΔCt) using the formula 2^-ΔCt^. Determination of circulating miRNA profiles included plasma samples from six-seven animals per group and a total of four experimental groups were integrated: 2–3 months old-3xTg-AD mice (*n* = 3), 2–3 months old-WT mice (*n* = 3), 14–15 months old-3xTg-AD (*n* = 3), and 14–15 months old-WT mice (*n* = 3) (*n* = samples).

When comparing the plasma of 14–15 months old 3xTg-AD mice vs. age-matched WT mice, we detected a significant lower abundance of miR-132, miR-138, miR-139, miR-146a, miR-146b, miR-22, miR-24, miR-29a, and miR-29c as well as a higher abundance of miR-346 (Figure 4, Supplementary Table 4). This finding suggests that the relative abundance of these miRNAs was altered specifically in the 3xTg-AD mice only at age 14–15 months since they were not altered at 2–3 months of age. These results correlate with the appearance of the histopathological features (Figure 1).

## Discussion

AD is a neurodegenerative disorder highly related to aging that exhibits progressive manifestations at both clinical and histopathological levels. The evolution of AD neuropathological features in the 3xTg-AD is also age-dependent (Oddo et al., 2003). In this work, we evaluated the levels of circulating miRNAs in four groups of animals: young WT; young 3xTg-AD; old WT and; old 3xTg-AD. For this purpose, we pooled plasma from two different animals from the same group and conformed three samples per group. Obtaining sufficient plasma for miRNA isolation can be problematic with young animals and getting sufficient animals with the triple transgene is difficult especially with old animals, since their health declines rapidly and mortality increases. Pooling the samples on the one hand, may pose a limitation given that each sample contains plasma from two different animals. But on the other hand, this design allowed us to avoid the bias from changes in circulating miRNAs associated to each individual. Even when the 3xTg-AD already bears the pathological transgenes, we did not find changes in the circulating miRNA profile between WT and 3xTg-AD young mice. This suggests that the presence of the transgenes is not sufficient to modify the circulating levels of miRNAs at early stages of the pathology. Nevertheless, we found age-related changes in the plasmatic abundance of certain miRNAs in both WT and 3xTg-AD mice. Remarkably we found changes in specific circulating miRNAs in the aging AD model. In agreement with other studies (Grillari et al., 2010; Kato et al., 2011; Martinez et al., 2011) we found a decrease in abundance of members of the family of let-7, miR-30, miR-17-92 cluster and its paralogs miR-106a-363 and miR-106b-25 in WT and 3x-Tg-AD aged mice. Let-7 family is highly involved in developmental processes in multiple species; it is required for cell differentiation thus its levels increase in late developmental stages and remain high in the adult (Thomson et al., 2004). Accordingly, let-7 family members target important cell cycle molecules (Schultz et al., 2008). miR-17-92 cluster and its paralogs are other important cell cycle modulators; interestingly, they are up-regulated in cancer and suppression of these clusters' members induce cell growth arrest in cancer models, whereas they are down-regulated in aging and their overexpression induces cellular senescence (Grillari et al., 2010).

In addition, we found some miRNAs involved with an inflammatory response such as miR-146a and miR-146b that display altered levels in plasma of both groups of old mice. This is particularly relevant considering that it has been established that inflammation is a condition closely related to the aging process. miR-146a and 146b are NF-κ B sensitive-miRNAs that suppress the immune response by inhibiting the pro-inflamatory immune cell signaling (Taganov et al., 2006). Interestingly, miR-146a displays a higher abundance in the 14–15-month old WT and a lower abundance in the 14–15-month 3xTg-AD as compared to their respective group of young mice. These bidirectional modulation agrees with recent data indicating that miR-146a is elevated in senescence models (Olivieri et al., 2013; Rippo et al., 2014), while it is decreased in AD (Kiko et al., 2014; Müller et al., 2014). A similar case is miR-34a, which is present with lower abundance in the 14–15-month 3xTg-AD mice as compared to the 2–3-month 3xTg-AD while showing a trend to increase with age in the WT. Higher levels of this miRNA are found in the brain, blood cells and plasma of old mice (Li et al., 2011) but the loss of miR-34a induces neurodegenerative phenotypes in *Drosophila*, and its overexpression induces longevity (Liu et al., 2012). Remarkably, lower levels of miR-34a have been reported in AD cases (Kiko et al., 2014; Müller et al., 2014).

Thus, it is evident that the age-dependent evolution of AD-like pathology produces specific changes in the circulating miRNA profile of the 3xTg-AD mice. As a consequence, we identified differences in the abundance of 10 miRNAs when comparing plasma samples of the 14–15- month 3xTg-AD with the 14–15-month WT mice. Most of the alterations observed in the aged 3xTg-AD mice consist on the reduction of plasmatic miRNAs levels as compared to the aged WT. This is in agreement with previous reports from brain, CSF and serum/plasma samples of AD patients where miR-15, miR-29, miR-101, miR-106, miR-107, and miR-181 have been shown to be diminished (Cogswell et al., 2008; Hébert et al., 2008; Wang et al., 2008b, 2011; Geekiyanage and Chan, 2011; Geekiyanage et al., 2011; Kumar et al., 2013; Tan et al., 2013; Burgos et al., 2014; Kiko et al., 2014).

Some of the 10 AD-like pathology-related miRNAs identified in this study have been previously associated with AD in different studies (Table 1). For instance, the miR-29 cluster is decreased in brain samples of AD patients, which correlates with an increase of the levels of the β-sercretase protein. Furthermore, members of this cluster have been shown to regulate the levels of the β-secretase *in vitro* (Hébert et al., 2008). Levels of miR-146a and miR-146b have been reported diminished in brain, CFS and plasma of AD patients (Cogswell et al., 2008; Kiko et al., 2014; Müller et al., 2014). These miRNAs are related to the inflammatory response, and may constitute an interesting result since neuroinflammation is a prevalent and early pathological feature of AD (Lukiw et al., 2008). In agreement with our results, lower levels of miR-132, miR-138 y miR-139 have been reported in AD brain and CFS samples (Cogswell et al., 2008; Burgos et al., 2014). miR-132 has been reported to have an anti-inflammatory effect, blocking acetylcholinesterase, elevating acetylcholine levels and consequently blocking NF-kB (Shaked et al., 2009). In neurons, miR-132 and miR-138 are expressed in response to synaptic activity and have a role in the modulation of morphologic events of neuroplasticity taking place in memory and cognition processes (Wayman et al., 2008; Siegel et al., 2009; Edbauer et al., 2010; Impey et al., 2010; Hansen et al., 2012; Bicker et al., 2014). Synapses represent vulnerable structures in AD and several pathologic molecular events take place in these sites comprising their function (Selkoe, 2002). Since memory and cognition are highly impaired in AD, the lower abundance of miR-132 and miR-138 result very interesting for analyzing the evolution of AD at a molecular level. It would be therefore interesting to determine changes in plasmatic miRNAs with the status of patients already showing mild cognitive impairment in order to advance the early diagnosis of AD.

**Table 1:** **AD-like pathology-related miRNAs identified in this study and previously associated with AD**.

**This study**			**Previous studies**			
**miRNA**	**Sample**	**Lower/Higher**	**Sample**	**Lower/Higher**	**References**	**Target**
miR-132	Plasma of 3xTg-AD and WT mice of 2–3 and 14–15 months	−	AD brain	−	Cogswell et al., 2008	p250-GAP
			AD neocortex	−	Hébert et al., 2013	
			AD CSF	−	Burgos et al., 2014	
miR-138		−	AD CSF	−	Burgos et al., 2014	APT1
miR-139		−	AD CSF	−	Burgos et al., 2014	
miR-146a		−	AD CSF/plasma	−	Kiko et al., 2014	IRAK-1 TRAF6
			AD CFS	−	Müller et al., 2014	
			AD hippocampus	±		
miR-146b		−	AD CSF	−	Cogswell et al., 2008	
			AD brain	−		
miR-29a		−	AD cortex	−	Hébert et al., 2008	BACE1
			AD serum	−	Geekiyanage et al., 2011	
			AD CSF	+	Kiko et al., 2014	
miR-29c		−	AD cortex	−	Hébert et al., 2008	

*The table contains data obtained from this study (left) and collected from others (right). It indicates the miRNA, the sample used for determination, the abundance respect to the control cases and the reference of the other studies. It also contains the mRNA-target for the miRNA: p250-GAP, a brain-enriched GTPase-activating protein for Rho Family GTPases involved in the N-Methyl-d-aspartate receptor (NMDAR) signaling; APT1, acyl protein thioesterase 1, an enzyme regulating the palmitoylation status of proteins that are known to function at the synapse; IRAK1, interleukin-1 receptor-associated kinase 1, a kinase that associates with the interleukin-1 receptor upon stimulation which is responsible for interleukin-1 transcription of NFkB; TRAF6, TNF receptor associated factor 6 involved in the regulation of inflammation response and apoptosis; BACE1, β-secretase, a protease that cleaves the APP in the β site to produce the Aβ peptide in the amyloidogenic APP processing*.

In this work we used a pre-designed miRNA microarray platform, which included a restricted number of miRNAs previously associated to different neuropathologies. Other miRNAs not included in this design may be associated also to AD. However, and since to our knowledge this is the first study of circulating miRNAs in this transgenic model, we decided to incorporate miRNAs that have been already associated to several neuropathologies. It is clear that given the multifactorial nature of the disease, it is likely that not only a single biomarker will meet the needs for clinical diagnosis. Here we propose that a relative simple, non-invasive procedure may provide useful information about the AD pathophysiology, detection, and evolution. Thus, combining a panel of miRNA detection with additional biomarkers may increase the sensitivity and specificity for early AD diagnosis.

## Conclusion

The aim of this study was to identify changes in the circulating miRNA profile of a transgenic mouse model of AD in two different stages of the evolution of AD-like pathology. Using these two time-windows it was not possible to detect early modifications in miRNA levels associated with prodromal AD. However, we distinguish variations in different miRNAs once the AD-like pathology is established which advances the molecular biomarkers field. Most of the miRNAs we found as potentially interesting biomarkers for AD have no identified targets that may be assoiciated to the disease and their role in the pathological mechanisms taking place in AD remains unknown. Our results however shed light on subtle molecular modifications associated to pathological aging and open new venues for studying the role of particular circulating miRNAs in the evolution of AD.

### Conflict of interest statement

The Guest Associate Editor Rosalinda Guevara-Guzmán declares that, despite being affiliated to the same institution as authors Sylvia Garza-Manero, Clorinda Arias, Federico Bermúdez-Rattoni, Luis Vaca and Angélica Zepeda, the review process was handled objectively and no conflict of interest exists. The authors declare that the research was conducted in the absence of any commercial or financial relationships that could be construed as a potential conflict of interest.

## References

[B1] Alexandrov P. N. Dua P. Hill J. M. Bhattacharjee S. Zhao Y. Lukiw W. J. (2012). microRNA (miRNA) speciation in Alzheimer's disease (AD) cerebrospinal fluid (CSF) and extracellular fluid (ECF). Int. J. Biochem. Mol. Biol. 3, 365–373. 23301201 PMC3533883

[B2] Alkalay A. Rabinovici G. D. Zimmerman G. Agarwal N. Kaufer D. Miller B. L.. (2013). Plasma acetylcholinesterase activity correlates with intracerebral β-amyloid load. Curr. Alzheimer Res. 10, 48–56. 10.2174/1567205011310010007 23157337 PMC3768143

[B3] Ameres S. L. Zamore P. D. (2013). Diversifying microRNA sequence and function. Nat. Rev. Mol. Cell Biol. 14, 475–488. 10.1038/nrm3611 23800994

[B4] Arroyo J. D. Chevillet J. R. Kroh E. M. Ruf I. K. Pritchard C. C. Gibson D. F.. (2011). Argonaute2 complexes carry a population of circulating microRNAs independent of vesicles in human plasma. Proc. Natl. Acad. Sci. U.S.A. 108, 5003–5008. 10.1073/pnas.1019055108 21383194 PMC3064324

[B5] Ballard C. Gauthier S. Corbett A. Brayne C. Aarsland D. Jones E. (2011). Alzheimer's disease. Lancet. 377, 1019–1031. 10.1016/S0140-6736(10)61349-9 21371747

[B6] Bartel D. P. (2009). MicroRNAs: target recognition and regulatory functions. Cell 136, 215–233. 10.1016/j.cell.2009.01.002 19167326 PMC3794896

[B7] Beveridge N. J. Gardiner E. Carroll A. P. Tooney P. A. Cairns M. J. (2010). Schizophrenia is associated with an increase in cortical microRNA biogenesis. Mol. Psychiatry 15, 1176–1189. 10.1038/mp.2009.84 19721432 PMC2990188

[B8] Bicker S. Lackinger M. Weiss K. Schratt G. (2014). MicroRNA-132, -134, and -138: a microRNA troika rules in neuronal dendrites. Cell. Mol. Life Sci. 71, 3987–4005. 10.1007/s00018-014-1671-7 25008044 PMC11113804

[B9] Burgos K. Malenica I. Metpally R. Courtright A. Rakela B. Beach T.. (2014). Profiles of extracellular miRNA in cerebrospinal fluid and serum from patients with Alzheimer's and Parkinson's diseases correlate with disease status and features of pathology. PLoS ONE 9:e94839. 10.1371/journal.pone.0094839 24797360 PMC4010405

[B10] Chen X. Ba Y. Ma L. Cai X. Yin Y. Wang K.. (2008). Characterization of microRNAs in serum: a novel class of biomarkers for diagnosis of cancer and other diseases. Cell Res. 18, 997–1006. 10.1038/cr.2008.282 18766170

[B11] Cogswell J. P. Ward J. Taylor I. A. Waters M. Shi Y. Cannon B.. (2008). Identification of miRNA changes in Alzheimer's disease brain and CSF yields putative biomarkers and insights into disease pathways. J. Alzheimers. Dis. 14, 27–41. 18525125 10.3233/jad-2008-14103

[B12] Dekosky S. T. Scheff S. W. Styren S. D. (1996). Structural correlates of cognition in dementia: quantification and assessment of synapse change. Neurodegeneration 5, 417–421. 10.1006/neur.1996.0056 9117556

[B13] Edbauer D. Neilson J. R. Foster K. A. Wang C. F. Seeburg D. P. Batterton M. N.. (2010). Regulation of synaptic structure and function by FMRP-associated microRNAs miR-125b and miR-132. Neuron 65, 373–384. 10.1016/j.neuron.2010.01.005 20159450 PMC5018398

[B14] Fabian M. R. Sonenberg N. (2012). The mechanics of miRNA-mediated gene silencing: a look under the hood of miRISC. Nat. Struct. Mol. Biol. 19, 586–593. 10.1038/nsmb.2296 22664986

[B15] Geekiyanage H. Chan C. (2011). MicroRNA-137/181c regulates serine palmitoyltransferase and in turn amyloid beta, novel targets in sporadic Alzheimer's disease. J. Neurosci. 31, 14820–14830. 10.1523/JNEUROSCI.3883-11.2011 21994399 PMC3200297

[B16] Geekiyanage H. Jicha G. A. Nelson P. T. Chan C. (2011). Blood serum miRNA: non-invasive biomarkers for Alzheimer's disease. Exp. Neurol. 235, 491–496. 10.1016/j.expneurol.2011.11.026 22155483 PMC3361462

[B17] Giraldez A. J. Cinalli R. M. Glasner M. E. Enright A. J. Thomson J. M. Baskerville S.. (2005). MicroRNAs regulate brain morphogenesis in zebrafish. Science 308, 833–838. 10.1126/science.1109020 15774722

[B18] Goate A. Chartier-Harlin M. C. Mullan M. Brown J. Crawford F. Fidani L.. (1991). Segregation of a missense mutation in the amyloid precursor protein gene with familial Alzheimer's disease. Nature 349, 704–706. 10.1038/349704a0 1671712

[B19] Grillari J. Hackl M. Grillari-Voglauer R. (2010). miR-17-92 cluster: ups and downs in cancer and aging. Biogerontology 11, 501–506. 10.1007/s10522-010-9272-9 20437201 PMC2899009

[B20] Hansen K. F. Karelina K. Sakamoto K. Wayman G. A. Impey S. Obrietan K. (2012). miRNA-132: a dynamic regulator of cognitive capacity. Brain Struct. Funct. 218, 817–831. 10.1007/s00429-012-0431-4 22706759 PMC3508255

[B21] Hébert S. S. Horre K. Nicolai L. Papadopoulou A. S. Mandemakers W. Silahtaroglu A. N.. (2008). Loss of microRNA cluster miR-29a/b-1 in sporadic Alzheimer's disease correlates with increased BACE1/beta-secretase expression. Proc. Natl. Acad. Sci. U.S.A. 105, 6415–6420. 10.1073/pnas.0710263105 18434550 PMC2359789

[B21a] Hébert S. S. Wang W-X. Zhu Q. Nelson P. T. (2013). A study of small RNAs from cerebral neocortex of pathology-verified Alzheimer's disease, dementia with lewy bodies, hippocampal sclerosis, frontotemporal lobar dementia, and non-demented human controls. J. Alzheimer's Dis. 35, 335–348. 10.3233/JAD-122350 23403535 PMC3753694

[B22] Impey S. Davare M. Lesiak A. Fortin D. Ando H. Varlamova O.. (2010). An activity-induced microRNA controls dendritic spine formation by regulating Rac1-PAK signaling. Mol. Cell. Neurosci. 43, 146–156. 10.1016/j.mcn.2009.10.005 19850129 PMC2818337

[B23] Johnson R. Zuccato C. Belyaev N. D. Guest D. J. Cattaneo E. Buckley N. J. (2008). A microRNA-based gene dysregulation pathway in Huntington's disease. Neurobiol. Dis. 29, 438–445. 10.1016/j.nbd.2007.11.001 18082412

[B24] Kato M. Chen X. Inukai S. Zhao H. Slack F. J. (2011). Age-associated changes in expression of small, noncoding RNAs, including microRNAs, in C. elegans. RNA 17, 1804–1820. 10.1261/rna.2714411 21810936 PMC3185914

[B25] Kiko T. Nakagawa K. Tsuduki T. Furukawa K. Arai H. Miyazawa T. (2014). MicroRNAs in plasma and cerebrospinal fluid as potential markers for Alzheimer's disease. J. Alzheimers. Dis. 39, 253–259. 10.3233/JAD-130932 24157723

[B26] Kim J. Inoue K. Ishii J. Vanti W. B. Voronov S. V. Murchison E.. (2007). A MicroRNA feedback circuit in midbrain dopamine neurons. Science 317, 1220–1224. 10.1126/science.1140481 17761882 PMC2782470

[B27] Klunk W. E. Engler H. Nordberg A. Wang Y. Blomqvist G. Holt D. P.. (2004). Imaging brain amyloid in Alzheimer's disease with Pittsburgh Compound-B. Ann. Neurol. 55, 306–319. 10.1002/ana.20009 14991808

[B28] Kumar P. Dezso Z. Mackenzie C. Oestreicher J. Agoulnik S. Byrne M.. (2013). Circulating miRNA biomarkers for Alzheimer's disease. PLoS ONE 8:e69807. 10.1371/journal.pone.0069807 23922807 PMC3726785

[B29] Lawrie C. H. Gal S. Dunlop H. M. Pushkaran B. Liggins A. P. Pulford K.. (2008). Detection of elevated levels of tumour-associated microRNAs in serum of patients with diffuse large B-cell lymphoma. Br. J. Haematol. 141, 672–675. 10.1111/j.1365-2141.2008.07077.x 18318758

[B30] Leidinger P. Backes C. Deutscher S. Schmitt K. Mueller S. C. Frese K.. (2013). A blood based 12-miRNA signature of Alzheimer disease patients. Genome Biol. 14:R78. 10.1186/gb-2013-14-7-r78 23895045 PMC4053778

[B31] Levy-Lahad E. Wasco W. Poorkaj P. Romano D. M. Oshima J. Pettingell W. H.. (1995). Candidate gene for the chromosome 1 familial Alzheimer's disease locus. Science 269, 973–977. 10.1126/science.7638622 7638622

[B32] Li X. Khanna A. Li N. Wang E. (2011). Circulatory miR34a as an RNAbased, noninvasive biomarker for brain aging. Aging 3, 985–1002. 22064828 10.18632/aging.100371 PMC3229974

[B33] Liu N. Landreh M. Cao K. Abe M. Hendriks G. J. Kennerdell J. R.. (2012). The microRNA miR-34 modulates ageing and neurodegeneration in Drosophila. Nature 482, 519–523. 10.1038/nature10810 22343898 PMC3326599

[B34] Lukiw W. J. (2007). Micro-RNA speciation in fetal, adult and Alzheimer's disease hippocampus. Neuroreport 18, 297–300. 10.1097/WNR.0b013e3280148e8b 17314675

[B35] Lukiw W. J. Zhao Y. Cui J. G. (2008). An NF-kappaB-sensitive micro RNA-146a-mediated inflammatory circuit in Alzheimer disease and in stressed human brain cells. J. Biol. Chem. 283, 31315–31322. 10.1074/jbc.M805371200 18801740 PMC2581572

[B36] Martinez I. Cazalla D. Almstead L. L. Steitz J. A. Dimaio D. (2011). miR-29 and miR-30 regulate B-Myb expression during cellular senescence. Proc. Natl. Acad. Sci. U.S.A. 108, 522–527. 10.1073/pnas.1017346108 21187425 PMC3021067

[B37] Mitchell P. S. Parkin R. K. Kroh E. M. Fritz B. R. Wyman S. K. Pogosova-Agadjanyan E. L.. (2008). Circulating microRNAs as stable blood-based markers for cancer detection. Proc. Natl. Acad. Sci. U.S.A. 105, 10513–10518. 10.1073/pnas.0804549105 18663219 PMC2492472

[B38] Müller M. Kuiperij H. B. Claassen J. A. Kusters B. Verbeek M. M. (2014). MicroRNAs in Alzheimer's disease: differential expression in hippocampus and cell-free cerebrospinal fluid. Neurobiol. Aging 35, 152–158. 10.1016/j.neurobiolaging.2013.07.005 23962497

[B39] Nunez-Iglesias J. Liu C. C. Morgan T. E. Finch C. E. Zhou X. J. (2010). Joint genome-wide profiling of miRNA and mRNA expression in Alzheimer's disease cortex reveals altered miRNA regulation. PLoS ONE 5:e8898. 10.1371/journal.pone.0008898 20126538 PMC2813862

[B40] Oddo S. Caccamo A. Shepherd J. D. Murphy M. P. Golde T. E. Kayed R.. (2003). Triple-transgenic model of Alzheimer's disease with plaques and tangles: intracellular Abeta and synaptic dysfunction. Neuron 39, 409–421. 10.1016/S0896-6273(03)00434-3 12895417

[B41] Olivieri F. Lazzarini R. Recchioni R. Marcheselli F. Rippo M. R. Di Nuzzo S.. (2013). MiR-146a as marker of senescence-associated pro-inflammatory status in cells involved in vascular remodelling. Age 35, 1157–1172. 10.1007/s11357-012-9440-8 22692818 PMC3705128

[B42] Rippo M. R. Olivieri F. Monsurro V. Prattichizzo F. Albertini M. C. Procopio A. D. (2014). MitomiRs in human inflamm-aging: a hypothesis involving miR-181a, miR-34a and miR-146a. Exp. Gerontol. 56, 154–163. 10.1016/j.exger.2014.03.002 24607549

[B43] Rogaev E. I. Sherrington R. Rogaeva E. A. Levesque G. Ikeda M. Liang Y.. (1995). Familial Alzheimer's disease in kindreds with missense mutations in a gene on chromosome 1 related to the Alzheimer's disease type 3 gene. Nature 376, 775–778. 10.1038/376775a0 7651536

[B44] Schultz J. Lorenz P. Gross G. Ibrahim S. Kunz M. (2008). MicroRNA let-7b targets important cell cycle molecules in malignant melanoma cells and interferes with anchorage-independent growth. Cell Res. 18, 549–557. 10.1038/cr.2008.45 18379589

[B45] Selkoe D. J. (2001). Alzheimer's disease: genes, proteins, and therapy. Physiol. Rev. 81, 741–766. 11274343 10.1152/physrev.2001.81.2.741

[B46] Selkoe D. J. (2002). Alzheimer's disease is a synaptic failure. Science 298, 789–791. 10.1126/science.1074069 12399581

[B47] Shaked I. Meerson A. Wolf Y. Avni R. Greenberg D. Gilboa-Geffen A.. (2009). MicroRNA-132 potentiates cholinergic anti-inflammatory signaling by targeting acetylcholinesterase. Immunity 31, 965–973. 10.1016/j.immuni.2009.09.019 20005135

[B48] Sherrington R. Rogaev E. I. Liang Y. Rogaeva E. A. Levesque G. Ikeda M.. (1995). Cloning of a gene bearing missense mutations in early-onset familial Alzheimer's disease. Nature 375, 754–760. 10.1038/375754a0 7596406

[B49] Siegel G. Obernosterer G. Fiore R. Oehmen M. Bicker S. Christensen M.. (2009). A functional screen implicates microRNA-138-dependent regulation of the depalmitoylation enzyme APT1 in dendritic spine morphogenesis. Nat. Cell Biol. 11, 705–716. 10.1038/ncb1876 19465924 PMC3595613

[B50] Taganov K. D. Boldin M. P. Chang K. J. Baltimore D. (2006). NF-kappaB-dependent induction of microRNA miR-146, an inhibitor targeted to signaling proteins of innate immune responses. Proc. Natl. Acad. Sci. U.S.A. 103, 12481–12486. 10.1073/pnas.0605298103 16885212 PMC1567904

[B51] Tan L. Yu J. T. Liu Q. Y. Tan M. S. Zhang W. Hu N.. (2013). Circulating miR-125b as a biomarker of Alzheimer's disease. J. Neurol. Sci. 336, 52–56. 10.1016/j.jns.2013.10.002 24139697

[B52] Terry R. D. Masliah E. Salmon D. P. Butters N. Deteresa R. Hill R.. (1991). Physical basis of cognitive alterations in Alzheimer's disease: synapse loss is the major correlate of cognitive impairment. Ann. Neurol. 30, 572–580. 10.1002/ana.410300410 1789684

[B53] Thomson J. M. Parker J. Perou C. M. Hammond S. M. (2004). A custom microarray platform for analysis of microRNA gene expression. Nat. Methods 1, 47–53. 10.1038/nmeth704 15782152

[B54] Valadi H. Ekstrom K. Bossios A. Sjostrand M. Lee J. J. Lotvall J. O. (2007). Exosome-mediated transfer of mRNAs and microRNAs is a novel mechanism of genetic exchange between cells. Nat. Cell Biol. 9, 654–659. 10.1038/ncb1596 17486113

[B55] Vickers K. C. Palmisano B. T. Shoucri B. M. Shamburek R. D. Remaley A. T. (2011). MicroRNAs are transported in plasma and delivered to recipient cells by high-density lipoproteins. Nat. Cell Biol. 13, 423–433. 10.1038/ncb2210 21423178 PMC3074610

[B56] Wang G. Van Der Walt J. M. Mayhew G. Li Y. J. Zuchner S. Scott W. K.. (2008a). Variation in the miRNA-433 binding site of FGF20 confers risk for Parkinson disease by overexpression of alpha-synuclein. Am. J. Hum. Genet. 82, 283–289. 10.1016/j.ajhg.2007.09.021 18252210 PMC2427225

[B57] Wang W. X. Huang Q. Hu Y. Stromberg A. J. Nelson P. T. (2011). Patterns of microRNA expression in normal and early Alzheimer's disease human temporal cortex: white matter versus gray matter. Acta Neuropathol. 121, 193–205. 10.1007/s00401-010-0756-0 20936480 PMC3073518

[B58] Wang W. X. Rajeev B. W. Stromberg A. J. Ren N. Tang G. Huang Q.. (2008b). The expression of microRNA miR-107 decreases early in Alzheimer's disease and may accelerate disease progression through regulation of beta-site amyloid precursor protein-cleaving enzyme 1. J. Neurosci. 28, 1213–1223. 10.1523/JNEUROSCI.5065-07.2008 18234899 PMC2837363

[B59] Wayman G. A. Davare M. Ando H. Fortin D. Varlamova O. Cheng H. Y.. (2008). An activity-regulated microRNA controls dendritic plasticity by down-regulating p250GAP. Proc. Natl. Acad. Sci. U.S.A. 105, 9093–9098. 10.1073/pnas.0803072105 18577589 PMC2449370

[B60] Yoo A. S. Staahl B. T. Chen L. Crabtree G. R. (2009). MicroRNA-mediated switching of chromatin-remodelling complexes in neural development. Nature 460, 642–646. 10.1038/nature08139 19561591 PMC2921580

[B61] Zhao C. Sun G. Li S. Shi Y. (2009). A feedback regulatory loop involving microRNA-9 and nuclear receptor TLX in neural stem cell fate determination. Nat. Struct. Mol. Biol. 16, 365–371. 10.1038/nsmb.1576 19330006 PMC2667220

