# Supplementary material for: Identification of age- and disease-related alterations in circulating miRNAs in a mouse model of Alzheimer's disease
Source: Front Cell Neurosci. 2015 Feb 19;9:53. doi: 10.3389/fncel.2015.00053 (PMC4333818; doi:10.3389/fncel.2015.00053)
Supplement: Supplementary file 1 [file Table1.DOCX]

**Sup. Table 1**

Comparison of plasmatic levels of evaluated miRNAs from 2-3 months-old 3xTg-AD and WT mice reveals no significant differences in the circulating miRNA profile between young mice. The table indicates the miRNA, the average relative abundance and the respective standard deviation (s) for each group, the fold-change and the p-value derived from the “t-test”. Relative abundance was calculated through the difference of the threshold cycle (Ct) of each miRNA and the Ct of the miR-39 of *C. elegans* mimic (ΔCt) using the formula 2^-ΔCt^. Fold-changes between the 2-3 months 3xTg-AD and WT groups were calculated using the formula 2^-ΔΔCt^.

| miRNA | WT of 2-3 months | | 3xTg-AD of 2-3 months | | Fold-change | p-value  “t-test” |
| --- | --- | --- | --- | --- | --- | --- |
|  | Relative abundance | s | Relative abundance | s |  |  |
| mmu-let-7b-5p | 0.4798 | 0.1407 | 0.5906 | 0.2505 | 1.23 | 0.5409 |
| mmu-let-7c-5p | 0.5966 | 0.1234 | 0.7152 | 0.1762 | 1.20 | 0.3937 |
| mmu-let-7d-5p | 0.2414 | 0.0673 | 0.2480 | 0.0910 | 1.03 | 0.9235 |
| mmu-let-7e-5p | 0.3241 | 0.0643 | 0.3598 | 0.0619 | 1.11 | 0.5267 |
| mmu-let-7i-5p | 0.3898 | 0.0371 | 0.4815 | 0.1890 | 1.24 | 0.4559 |
| mmu-miR-101a-3p | 0.0655 | 0.0344 | 0.0820 | 0.0318 | 1.25 | 0.5752 |
| mmu-miR-101b-3p | 0.0806 | 0.0393 | 0.1091 | 0.0571 | 1.35 | 0.5155 |
| mmu-miR-105 | ND |  | ND |  |  |  |
| mmu-miR-106b-5p | 0.6494 | 0.0783 | 0.6731 | 0.0825 | 1.04 | 0.7365 |
| mmu-miR-107-3p | 0.0041 | 0.0013 | 0.0057 | 0.0019 | 1.38 | 0.3016 |
| mmu-miR-124-3p | 0.0255 | 0.0247 | 0.0089 | 0.0083 | 0.35 | 0.3334 |
| mmu-miR-125b-5p | 0.4047 | 0.1075 | 0.5762 | 0.2351 | 1.42 | 0.3146 |
| mmu-miR-126-5p | 0.3249 | 0.0742 | 0.3803 | 0.0095 | 1.17 | 0.2685 |
| mmu-miR-128-3p | 0.0464 | 0.0071 | 0.0539 | 0.0219 | 1.16 | 0.6025 |
| mmu-miR-130a-3p | 0.0889 | 0.0141 | 0.1118 | 0.0102 | 1.26 | 0.0853 |
| mmu-miR-132-3p | 0.0218 | 0.0053 | 0.0174 | 0.0040 | 0.80 | 0.3161 |
| mmu-miR-133b-3p | 0.0417 | 0.0278 | 0.0417 | 0.0302 | 1.00 | 0.9977 |
| mmu-miR-134-5p | 0.0027 | 0.0021 | 0.0046 | 0.0005 | 1.71 | 0.2012 |
| mmu-miR-135b-5p | ND |  | ND |  |  |  |
| mmu-miR-138-5p | 0.0177 | 0.0060 | 0.0157 | 0.0038 | 0.89 | 0.6476 |
| mmu-miR-139-5p | 0.0985 | 0.0298 | 0.1113 | 0.0362 | 1.13 | 0.6607 |
| mmu-miR-140-5p | 0.0498 | 0.0050 | 0.0542 | 0.0066 | 1.09 | 0.4030 |
| mmu-miR-146a-5p | 0.1664 | 0.0123 | 0.1829 | 0.0276 | 1.10 | 0.3957 |
| mmu-miR-146b-5p | 0.0522 | 0.0178 | 0.0595 | 0.0137 | 1.14 | 0.6052 |
| mmu-miR-148b-3p | 0.0463 | 0.0155 | 0.0507 | 0.0324 | 1.10 | 0.8419 |
| mmu-miR-151-3p | 0.0210 | 0.0037 | 0.0235 | 0.0075 | 1.12 | 0.6302 |
| mmu-miR-152-3p | 0.0358 | 0.0122 | 0.0542 | 0.0271 | 1.51 | 0.3434 |
| mmu-miR-15a-5p | 0.2008 | 0.0496 | 0.2475 | 0.1340 | 1.23 | 0.6018 |
| mmu-miR-15b-5p | 0.2464 | 0.0460 | 0.2579 | 0.0891 | 1.05 | 0.8522 |
| mmu-miR-181a-5p | 0.0431 | 0.0045 | 0.0633 | 0.0430 | 1.47 | 0.4646 |
| mmu-miR-181a1-3p | 0.0020 | 0.0004 | 0.0018 | 0.0010 | 0.87 | 0.7035 |
| mmu-miR-181c-5p | 0.0416 | 0.0031 | 0.0607 | 0.0413 | 1.46 | 0.4695 |
| mmu-miR-181d-5p | 0.0427 | 0.0125 | 0.0536 | 0.0255 | 1.26 | 0.5424 |
| mmu-miR-191-5p | 0.3962 | 0.0373 | 0.4758 | 0.2549 | 1.20 | 0.6207 |
| mmu-miR-193b-3p | 0.0367 | 0.0110 | 0.0475 | 0.0232 | 1.29 | 0.5060 |
| mmu-miR-194-5p | 0.2151 | 0.0346 | 0.1922 | 0.0669 | 0.89 | 0.6263 |
| mmu-miR-195a-5p | 5.1632 | 0.2950 | 4.9744 | 0.5750 | 0.96 | 0.6394 |
| mmu-miR-19b-3p | 0.4136 | 0.1126 | 0.4400 | 0.0390 | 1.06 | 0.7210 |
| mmu-miR-203-3p | 0.0842 | 0.0283 | 0.1334 | 0.0457 | 1.58 | 0.1881 |
| mmu-miR-20a-5p | 0.6911 | 0.0198 | 0.7275 | 0.0543 | 1.05 | 0.3371 |
| mmu-miR-20b-5p | 0.4203 | 0.0681 | 0.4158 | 0.0685 | 0.99 | 0.9404 |
| mmu-miR-22-3p | 2.0795 | 0.2852 | 2.5214 | 0.2784 | 1.21 | 0.1272 |
| mmu-miR-24-3p | 1.5914 | 0.2381 | 2.1088 | 0.5253 | 1.33 | 0.1951 |
| mmu-miR-26b-5p | 0.3846 | 0.0825 | 0.4535 | 0.0802 | 1.18 | 0.3579 |
| mmu-miR-27a-3p | 1.1968 | 0.1614 | 1.6693 | 0.4607 | 1.39 | 0.1689 |
| mmu-miR-28c | 0.0052 | 0.0018 | 0.0056 | 0.0019 | 1.08 | 0.7921 |
| mmu-miR-298-5p | 0.0038 | 0.0021 | 0.0068 | 0.0039 | 1.78 | 0.3080 |
| mmu-miR-29a-3p | 1.1202 | 0.1005 | 1.4424 | 0.3703 | 1.29 | 0.2195 |
| mmu-miR-29b-3p | 0.1271 | 0.0368 | 0.1726 | 0.0064 | 1.36 | 0.1023 |
| mmu-miR-29c-3p | 1.0896 | 0.0346 | 1.3157 | 0.1610 | 1.21 | 0.0761 |
| mmu-miR-302a-5p | ND |  | ND |  |  |  |
| mmu-miR-302b-5p | ND |  | ND |  |  |  |
| mmu-miR-30a-5p | 0.8702 | 0.0666 | 0.9987 | 0.2843 | 1.15 | 0.4887 |
| mmu-miR-30d-5p | 0.3581 | 0.0224 | 0.3707 | 0.1076 | 1.04 | 0.8531 |
| mmu-miR-30e-5p | 0.8556 | 0.0505 | 0.8850 | 0.1201 | 1.03 | 0.7153 |
| mmu-miR-320-3p | 0.1571 | 0.0438 | 0.1961 | 0.0647 | 1.25 | 0.4361 |
| mmu-miR-328-3p | 0.0890 | 0.0268 | 0.1185 | 0.0870 | 1.33 | 0.6047 |
| mmu-miR-33-5p | 0.0188 | 0.0217 | 0.0173 | 0.0067 | 0.92 | 0.9111 |
| mmu-miR-337-3p | 0.0071 | 0.0027 | 0.0090 | 0.0017 | 1.26 | 0.3776 |
| mmu-miR-338-3p | 0.0067 | 0.0012 | 0.0107 | 0.0022 | 1.59 | 0.0503 |
| mmu-miR-339-5p | 0.0602 | 0.0166 | 0.0705 | 0.0337 | 1.17 | 0.6576 |
| mmu-miR-342-3p | 0.2048 | 0.0478 | 0.2009 | 0.0843 | 0.98 | 0.9479 |
| mmu-miR-346-5p | 0.0013 | 0.0005 | 0.0024 | 0.0013 | 1.78 | 0.2700 |
| mmu-miR-34a-5p | 0.0155 | 0.0063 | 0.0179 | 0.0048 | 1.16 | 0.6239 |
| mmu-miR-376b-3p | 0.0031 | 0.0011 | 0.0066 | 0.0019 | 2.13 | 0.0519 |
| mmu-miR-381-3p | 0.0014 | 0.0006 | 0.0030 | 0.0009 | 2.16 | 0.0619 |
| mmu-miR-409-3p | 0.0026 | 0.0011 | 0.0027 | 0.0008 | 1.04 | 0.8998 |
| mmu-miR-431-5p | 0.0025 | 0.0012 | 0.0031 | 0.0004 | 1.26 | 0.4337 |
| mmu-miR-433-3p | 0.0022 | 0.0009 | 0.0040 | 0.0008 | 1.80 | 0.0595 |
| mmu-miR-455-5p | 0.0059 | 0.0022 | 0.0091 | 0.0021 | 1.54 | 0.1421 |
| mmu-miR-484 | 0.2867 | 0.0434 | 0.3775 | 0.1568 | 1.32 | 0.3886 |
| mmu-miR-485-5p | 0.0004 | 0.0002 | 0.0006 | 0.0001 | 1.43 | 0.2342 |
| mmu-miR-485-3p | 0.0022 | 0.0008 | 0.0036 | 0.0009 | 1.63 | 0.1213 |
| mmu-miR-488-3p | ND |  | ND |  |  |  |
| mmu-miR-489-3p | 0.0004 | 0.0004 | 0.0006 | 0.0003 | 1.63 | 0.4428 |
| mmu-miR-509-3p | ND |  | ND |  |  |  |
| mmu-miR-598-3p | 0.0015 | 0.0009 | 0.0027 | 0.0019 | 1.83 | 0.3714 |
| mmu-miR-652-3p | 0.0655 | 0.0158 | 0.0688 | 0.0277 | 1.05 | 0.8660 |
| mmu-miR-7a-5p | 0.0147 | 0.0031 | 0.0125 | 0.0008 | 0.85 | 0.2847 |
| mmu-miR-9-5p | 0.0011 | 0.0009 | 0.0004 | 0.0002 | 0.37 | 0.2963 |
| mmu-miR-9-3p | ND |  | ND |  |  |  |
| mmu-miR-92a-3p | 0.9485 | 0.2535 | 1.2146 | 0.6501 | 1.28 | 0.5450 |
| mmu-miR-93-5p | 0.6291 | 0.1129 | 0.6479 | 0.2671 | 1.03 | 0.9159 |
| mmu-miR-98-5p | 0.0073 | 0.0028 | 0.0061 | 0.0023 | 0.83 | 0.5904 |

**Sup. Table 2**

Comparison of plasmatic levels of evaluated miRNAs from WT mice of 2-3 and 14-15 months suggests age-related significant differences in the circulating miRNA profile between young and old mice. The table indicates the miRNA, the average relative abundance and its respective standard deviation (s) of each group, the fold-change and the p-value derived from the “t-test” probe. Relative abundances were calculated with the difference of the Ct of each miRNA and the Ct of the miR-39 of *C. elegans* mimics (ΔCt) using the formula 2^-ΔCt^. Fold-changes between the WT of 2-3 and 14-15 months groups were calculated with the formula 2^-ΔΔCt^.

| miRNA | WT of 2-3 months | | WT of 14-15 months | | Fold-change | p-value  “t-test” |
| --- | --- | --- | --- | --- | --- | --- |
|  | Relative abundance | s | Relative abundance | s |  |  |
| mmu-let-7b-5p | 0.4798 | 0.1407 | 0.2725 | 0.0343 | 0.57 | 0.0683 |
| mmu-let-7c-5p | 0.5966 | 0.1234 | 0.3838 | 0.0387 | 0.64 | **0.0464** |
| mmu-let-7d-5p | 0.2414 | 0.0673 | 0.0856 | 0.0243 | 0.35 | **0.0196** |
| mmu-let-7e-5p | 0.3241 | 0.0643 | 0.1476 | 0.0216 | 0.46 | **0.0108** |
| mmu-let-7i-5p | 0.3898 | 0.0371 | 0.1858 | 0.0575 | 0.48 | **0.0067** |
| mmu-miR-101a-3p | 0.0655 | 0.0344 | 0.0476 | 0.0246 | 0.73 | 0.5039 |
| mmu-miR-101b-3p | 0.0806 | 0.0393 | 0.0538 | 0.0301 | 0.67 | 0.4005 |
| mmu-miR-105 | ND |  | ND |  |  |  |
| mmu-miR-106b-5p | 0.6494 | 0.0783 | 0.3498 | 0.1147 | 0.54 | **0.0202** |
| mmu-miR-107-3p | 0.0041 | 0.0013 | 0.0027 | 0.0007 | 0.65 | 0.1497 |
| mmu-miR-124-3p | 0.0255 | 0.0247 | 0.0179 | 0.0156 | 0.70 | 0.6750 |
| mmu-miR-125b-5p | 0.4047 | 0.1075 | 0.2427 | 0.0549 | 0.60 | 0.0808 |
| mmu-miR-126-5p | 0.3249 | 0.0742 | 0.1754 | 0.0495 | 0.54 | **0.0440** |
| mmu-miR-128-3p | 0.0464 | 0.0071 | 0.0389 | 0.0080 | 0.84 | 0.2978 |
| mmu-miR-130a-3p | 0.0889 | 0.0141 | 0.0569 | 0.0060 | 0.64 | **0.0226** |
| mmu-miR-132-3p | 0.0218 | 0.0053 | 0.0160 | 0.0017 | 0.73 | 0.1443 |
| mmu-miR-133b-3p | 0.0417 | 0.0278 | 0.0230 | 0.0220 | 0.55 | 0.4134 |
| mmu-miR-134-5p | 0.0027 | 0.0021 | 0.0005 | 0.0002 | 0.19 | 0.1509 |
| mmu-miR-135b-5p | ND |  | ND |  |  |  |
| mmu-miR-138-5p | 0.0177 | 0.0060 | 0.0160 | 0.0048 | 0.90 | 0.7196 |
| mmu-miR-139-5p | 0.0985 | 0.0298 | 0.0904 | 0.0054 | 0.92 | 0.6656 |
| mmu-miR-140-5p | 0.0498 | 0.0050 | 0.0221 | 0.0048 | 0.44 | **0.0023** |
| mmu-miR-146a-5p | 0.1664 | 0.0123 | 0.2004 | 0.0101 | 1.20 | **0.0205** |
| mmu-miR-146b-5p | 0.0522 | 0.0178 | 0.0645 | 0.0080 | 1.23 | 0.3394 |
| mmu-miR-148b-3p | 0.0463 | 0.0155 | 0.0244 | 0.0116 | 0.53 | 0.1223 |
| mmu-miR-151-3p | 0.0210 | 0.0037 | 0.0142 | 0.0016 | 0.68 | **0.0452** |
| mmu-miR-152-3p | 0.0358 | 0.0122 | 0.0193 | 0.0077 | 0.54 | 0.1199 |
| mmu-miR-15a-5p | 0.2008 | 0.0496 | 0.0568 | 0.0304 | 0.28 | **0.0128** |
| mmu-miR-15b-5p | 0.2464 | 0.0460 | 0.1333 | 0.0428 | 0.54 | **0.0357** |
| mmu-miR-181a-5p | 0.0431 | 0.0045 | 0.0168 | 0.0041 | 0.39 | **0.0017** |
| mmu-miR-181a1-3p | 0.0020 | 0.0004 | 0.0005 | 0.0002 | 0.24 | **0.0036** |
| mmu-miR-181c-5p | 0.0416 | 0.0031 | 0.0152 | 0.0043 | 0.36 | **0.0010** |
| mmu-miR-181d-5p | 0.0427 | 0.0125 | 0.0200 | 0.0024 | 0.47 | **0.0360** |
| mmu-miR-191-5p | 0.3962 | 0.0373 | 0.2947 | 0.0483 | 0.74 | **0.0450** |
| mmu-miR-193b-3p | 0.0367 | 0.0110 | 0.0168 | 0.0023 | 0.46 | **0.0378** |
| mmu-miR-194-5p | 0.2151 | 0.0346 | 0.0892 | 0.0241 | 0.41 | **0.0067** |
| mmu-miR-195a-5p | 5.1632 | 0.2950 | 2.1263 | 0.2812 | 0.41 | **0.0002** |
| mmu-miR-19b-3p | 0.4136 | 0.1126 | 0.3347 | 0.1282 | 0.81 | 0.4678 |
| mmu-miR-203-3p | 0.0842 | 0.0283 | 0.1242 | 0.0413 | 1.48 | 0.2383 |
| mmu-miR-20a-5p | 0.6911 | 0.0198 | 0.4233 | 0.1385 | 0.61 | **0.0295** |
| mmu-miR-20b-5p | 0.4203 | 0.0681 | 0.1918 | 0.0474 | 0.46 | **0.0088** |
| mmu-miR-22-3p | 2.0795 | 0.2852 | 1.6335 | 0.1637 | 0.79 | 0.0786 |
| mmu-miR-24-3p | 1.5914 | 0.2381 | 1.4870 | 0.1634 | 0.93 | 0.5654 |
| mmu-miR-26b-5p | 0.3846 | 0.0825 | 0.1623 | 0.0680 | 0.42 | **0.0227** |
| mmu-miR-27a-3p | 1.1968 | 0.1614 | 1.3101 | 0.4461 | 1.09 | 0.7004 |
| mmu-miR-28c | 0.0052 | 0.0018 | 0.0020 | 0.0007 | 0.38 | **0.0468** |
| mmu-miR-298-5p | 0.0038 | 0.0021 | 0.0009 | 0.0002 | 0.23 | 0.0701 |
| mmu-miR-29a-3p | 1.1202 | 0.1005 | 1.0110 | 0.1881 | 0.90 | 0.4250 |
| mmu-miR-29b-3p | 0.1271 | 0.0368 | 0.0828 | 0.0269 | 0.65 | 0.1678 |
| mmu-miR-29c-3p | 1.0896 | 0.0346 | 0.9583 | 0.1753 | 0.88 | 0.2721 |
| mmu-miR-302a-5p | ND |  | ND |  |  |  |
| mmu-miR-302b-5p | ND |  | ND |  |  |  |
| mmu-miR-30a-5p | 0.8702 | 0.0666 | 0.5814 | 0.0252 | 0.67 | **0.0022** |
| mmu-miR-30d-5p | 0.3581 | 0.0224 | 0.2527 | 0.0338 | 0.71 | **0.0108** |
| mmu-miR-30e-5p | 0.8556 | 0.0505 | 0.5464 | 0.0544 | 0.64 | **0.0020** |
| mmu-miR-320-3p | 0.1571 | 0.0438 | 0.1075 | 0.0190 | 0.68 | 0.1462 |
| mmu-miR-328-3p | 0.0890 | 0.0268 | 0.0658 | 0.0188 | 0.74 | 0.2878 |
| mmu-miR-33-5p | 0.0188 | 0.0217 | 0.0082 | 0.0058 | 0.43 | 0.4575 |
| mmu-miR-337-3p | 0.0071 | 0.0027 | 0.0016 | 0.0007 | 0.22 | **0.0266** |
| mmu-miR-338-3p | 0.0067 | 0.0012 | 0.0073 | 0.0030 | 1.09 | 0.7619 |
| mmu-miR-339-5p | 0.0602 | 0.0166 | 0.0404 | 0.0082 | 0.67 | 0.1374 |
| mmu-miR-342-3p | 0.2048 | 0.0478 | 0.1719 | 0.0526 | 0.84 | 0.4672 |
| mmu-miR-346-5p | 0.0013 | 0.0005 | 0.0006 | 0.0002 | 0.41 | 0.0548 |
| mmu-miR-34a-5p | 0.0155 | 0.0063 | 0.0167 | 0.0048 | 1.08 | 0.8065 |
| mmu-miR-376b-3p | 0.0031 | 0.0011 | 0.0016 | 0.0007 | 0.50 | 0.1097 |
| mmu-miR-381-3p | 0.0014 | 0.0006 | 0.0006 | 0.0005 | 0.40 | 0.1349 |
| mmu-miR-409-3p | 0.0026 | 0.0011 | 0.0008 | 0.0003 | 0.30 | **0.0456** |
| mmu-miR-431-5p | 0.0025 | 0.0012 | 0.0011 | 0.0006 | 0.46 | 0.1555 |
| mmu-miR-433-3p | 0.0022 | 0.0009 | 0.0021 | 0.0007 | 0.92 | 0.7956 |
| mmu-miR-455-5p | 0.0059 | 0.0022 | 0.0014 | 0.0001 | 0.24 | **0.0248** |
| mmu-miR-484 | 0.2867 | 0.0434 | 0.2823 | 0.0651 | 0.98 | 0.9258 |
| mmu-miR-485-5p | 0.0004 | 0.0002 | 0.0002 | 0.0001 | 0.49 | 0.1472 |
| mmu-miR-485-3p | 0.0022 | 0.0008 | 0.0011 | 0.0001 | 0.47 | 0.0753 |
| mmu-miR-488-3p | ND |  | ND |  |  |  |
| mmu-miR-489-3p | 0.0004 | 0.0004 | ND |  |  |  |
| mmu-miR-509-3p | ND |  | ND |  |  |  |
| mmu-miR-598-3p | 0.0015 | 0.0009 | 0.0013 | 0.0003 | 0.89 | 0.7795 |
| mmu-miR-652-3p | 0.0655 | 0.0158 | 0.0492 | 0.0088 | 0.75 | 0.1955 |
| mmu-miR-7a-5p | 0.0147 | 0.0031 | 0.0074 | 0.0013 | 0.50 | **0.0193** |
| mmu-miR-9-5p | 0.0011 | 0.0009 | ND |  |  |  |
| mmu-miR-9-3p | ND |  | ND |  |  |  |
| mmu-miR-92a-3p | 0.9485 | 0.2535 | 0.6599 | 0.1835 | 0.70 | 0.1854 |
| mmu-miR-93-5p | 0.6291 | 0.1129 | 0.3036 | 0.0576 | 0.48 | **0.0112** |
| mmu-miR-98-5p | 0.0073 | 0.0028 | 0.0017 | 0.0010 | 0.23 | **0.0302** |

**Sup. Table 3**

Comparison of plasmatic levels of evaluated miRNAs from 3xTg-AD mice of 2-3 and 14-15 months suggests age-related significant differences in the circulating miRNA profile between young and old mice, including some specific age-related changes in the transgenic mice. The table indicates the miRNA, the average relative abundance and the respective standard deviation (s) for each group, the fold-change and the p-value derived from the “t-test”. Relative abundance was calculated through the difference of the of the threshold cycle (Ct) of each miRNA and the Ct of the miR-39 of *C. elegans* mimic (ΔCt) using the formula 2^-ΔCt^. Fold-changes between the 3xTg-AD of 2-3 and 14-15 months groups were calculated with the formula 2^-ΔΔCt^.

| miRNA | 3xTg-AD of 2-3 months | | 3xTg-AD of 14-15 months | | Fold-change | p-value  “t-test” |
| --- | --- | --- | --- | --- | --- | --- |
|  | Relative abundance | s | Relative abundance | s |  |  |
| mmu-let-7b-5p | 0.5906 | 0.2505 | 0.3016 | 0.0648 | 0.51 | 0.1252 |
| mmu-let-7c-5p | 0.7152 | 0.1762 | 0.3344 | 0.0919 | 0.47 | **0.0294** |
| mmu-let-7d-5p | 0.2480 | 0.0910 | 0.0935 | 0.0403 | 0.38 | 0.0546 |
| mmu-let-7e-5p | 0.3598 | 0.0619 | 0.1461 | 0.0522 | 0.41 | **0.0102** |
| mmu-let-7i-5p | 0.4815 | 0.1890 | 0.1662 | 0.0540 | 0.35 | **0.0499** |
| mmu-miR-101a-3p | 0.0820 | 0.0318 | 0.0240 | 0.0076 | 0.29 | **0.0374** |
| mmu-miR-101b-3p | 0.1091 | 0.0571 | 0.0250 | 0.0064 | 0.23 | 0.0642 |
| mmu-miR-105 | ND |  | ND |  |  |  |
| mmu-miR-106b-5p | 0.6731 | 0.0825 | 0.3346 | 0.0884 | 0.50 | **0.0083** |
| mmu-miR-107-3p | 0.0057 | 0.0019 | 0.0026 | 0.0011 | 0.45 | 0.0724 |
| mmu-miR-124-3p | 0.0089 | 0.0083 | 0.0034 | 0.0020 | 0.38 | 0.3257 |
| mmu-miR-125b-5p | 0.5762 | 0.2351 | 0.1617 | 0.0199 | 0.28 | **0.0383** |
| mmu-miR-126-5p | 0.3803 | 0.0095 | 0.1408 | 0.0486 | 0.37 | **0.0011** |
| mmu-miR-128-3p | 0.0539 | 0.0219 | 0.0362 | 0.0059 | 0.67 | 0.2491 |
| mmu-miR-130a-3p | 0.1118 | 0.0102 | 0.0626 | 0.0257 | 0.56 | **0.0369** |
| mmu-miR-132-3p | 0.0174 | 0.0040 | 0.0077 | 0.0004 | 0.45 | **0.0141** |
| mmu-miR-133b-3p | 0.0417 | 0.0302 | 0.0178 | 0.0098 | 0.43 | 0.2609 |
| mmu-miR-134-5p | 0.0046 | 0.0005 | 0.0008 | 0.0003 | 0.17 | **0.0003** |
| mmu-miR-135b-5p | ND |  | ND |  |  |  |
| mmu-miR-138-5p | 0.0157 | 0.0038 | 0.0076 | 0.0004 | 0.48 | **0.0214** |
| mmu-miR-139-5p | 0.1113 | 0.0362 | 0.0616 | 0.0100 | 0.55 | 0.0835 |
| mmu-miR-140-5p | 0.0542 | 0.0066 | 0.0243 | 0.0082 | 0.45 | **0.0079** |
| mmu-miR-146a-5p | 0.1829 | 0.0276 | 0.0910 | 0.0188 | 0.50 | **0.0089** |
| mmu-miR-146b-5p | 0.0595 | 0.0137 | 0.0272 | 0.0085 | 0.46 | **0.0255** |
| mmu-miR-148b-3p | 0.0507 | 0.0324 | 0.0160 | 0.0048 | 0.32 | 0.1399 |
| mmu-miR-151-3p | 0.0235 | 0.0075 | 0.0122 | 0.0019 | 0.52 | 0.0656 |
| mmu-miR-152-3p | 0.0542 | 0.0271 | 0.0117 | 0.0040 | 0.22 | 0.0547 |
| mmu-miR-15a-5p | 0.2475 | 0.1340 | 0.0719 | 0.0263 | 0.29 | 0.0900 |
| mmu-miR-15b-5p | 0.2579 | 0.0891 | 0.1032 | 0.0390 | 0.40 | 0.0512 |
| mmu-miR-181a-5p | 0.0633 | 0.0430 | 0.0172 | 0.0031 | 0.27 | 0.1377 |
| mmu-miR-181a1-3p | 0.0018 | 0.0010 | 0.0005 | 0.0003 | 0.28 | 0.1053 |
| mmu-miR-181c-5p | 0.0607 | 0.0413 | 0.0162 | 0.0074 | 0.27 | 0.1404 |
| mmu-miR-181d-5p | 0.0536 | 0.0255 | 0.0194 | 0.0045 | 0.36 | 0.0838 |
| mmu-miR-191-5p | 0.4758 | 0.2549 | 0.2818 | 0.0393 | 0.59 | 0.2624 |
| mmu-miR-193b-3p | 0.0475 | 0.0232 | 0.0193 | 0.0026 | 0.41 | 0.1043 |
| mmu-miR-194-5p | 0.1922 | 0.0669 | 0.0740 | 0.0267 | 0.38 | **0.0467** |
| mmu-miR-195a-5p | 4.9744 | 0.5750 | 2.2807 | 0.6503 | 0.46 | **0.0058** |
| mmu-miR-19b-3p | 0.4400 | 0.0390 | 0.2562 | 0.0557 | 0.58 | **0.0094** |
| mmu-miR-203-3p | 0.1334 | 0.0457 | 0.0849 | 0.0410 | 0.64 | 0.2435 |
| mmu-miR-20a-5p | 0.7275 | 0.0543 | 0.3740 | 0.1224 | 0.51 | **0.0103** |
| mmu-miR-20b-5p | 0.4158 | 0.0685 | 0.1778 | 0.0717 | 0.43 | **0.0142** |
| mmu-miR-22-3p | 2.5214 | 0.2784 | 1.2351 | 0.1543 | 0.49 | **0.0022** |
| mmu-miR-24-3p | 2.1088 | 0.5253 | 1.0586 | 0.1150 | 0.50 | **0.0277** |
| mmu-miR-26b-5p | 0.4535 | 0.0802 | 0.1234 | 0.0246 | 0.27 | **0.0024** |
| mmu-miR-27a-3p | 1.6693 | 0.4607 | 0.6975 | 0.1204 | 0.42 | **0.0241** |
| mmu-miR-28c | 0.0056 | 0.0019 | 0.0018 | 0.0006 | 0.31 | **0.0290** |
| mmu-miR-298-5p | 0.0068 | 0.0039 | 0.0033 | 0.0023 | 0.48 | 0.2482 |
| mmu-miR-29a-3p | 1.4424 | 0.3703 | 0.6036 | 0.0613 | 0.42 | **0.0180** |
| mmu-miR-29b-3p | 0.1726 | 0.0064 | 0.0637 | 0.0078 | 0.37 | **0.00005** |
| mmu-miR-29c-3p | 1.3157 | 0.1610 | 0.5189 | 0.0609 | 0.39 | **0.0013** |
| mmu-miR-302a-5p | ND |  | ND |  |  |  |
| mmu-miR-302b-5p | ND |  | ND |  |  |  |
| mmu-miR-30a-5p | 0.9987 | 0.2843 | 0.5054 | 0.0540 | 0.51 | **0.0419** |
| mmu-miR-30d-5p | 0.3707 | 0.1076 | 0.2177 | 0.0321 | 0.59 | 0.0778 |
| mmu-miR-30e-5p | 0.8850 | 0.1201 | 0.4927 | 0.0518 | 0.56 | **0.0065** |
| mmu-miR-320-3p | 0.1961 | 0.0647 | 0.0936 | 0.0060 | 0.48 | 0.0522 |
| mmu-miR-328-3p | 0.1185 | 0.0870 | 0.0781 | 0.0081 | 0.66 | 0.4688 |
| mmu-miR-33-5p | 0.0173 | 0.0067 | 0.0062 | 0.0016 | 0.36 | 0.0510 |
| mmu-miR-337-3p | 0.0090 | 0.0017 | 0.0018 | 0.0008 | 0.20 | **0.0029** |
| mmu-miR-338-3p | 0.0107 | 0.0022 | 0.0056 | 0.0022 | 0.52 | **0.0481** |
| mmu-miR-339-5p | 0.0705 | 0.0337 | 0.0486 | 0.0091 | 0.69 | 0.3384 |
| mmu-miR-342-3p | 0.2009 | 0.0843 | 0.1109 | 0.0093 | 0.55 | 0.1397 |
| mmu-miR-346-5p | 0.0024 | 0.0013 | 0.0016 | 0.0006 | 0.69 | 0.4292 |
| mmu-miR-34a-5p | 0.0179 | 0.0048 | 0.0085 | 0.0024 | 0.47 | **0.0388** |
| mmu-miR-376b-3p | 0.0066 | 0.0019 | 0.0010 | 0.0001 | 0.16 | **0.0072** |
| mmu-miR-381-3p | 0.0030 | 0.0009 | 0.0007 | 0.0001 | 0.22 | **0.0108** |
| mmu-miR-409-3p | 0.0027 | 0.0008 | 0.0009 | 0.0002 | 0.33 | **0.0195** |
| mmu-miR-431-5p | 0.0031 | 0.0004 | 0.0013 | 0.0004 | 0.43 | **0.0072** |
| mmu-miR-433-3p | 0.0040 | 0.0008 | 0.0023 | 0.0003 | 0.58 | **0.0208** |
| mmu-miR-455-5p | 0.0091 | 0.0021 | 0.0012 | 0.0006 | 0.13 | **0.0033** |
| mmu-miR-484 | 0.3775 | 0.1568 | 0.2338 | 0.0204 | 0.62 | 0.1907 |
| mmu-miR-485-5p | 0.0006 | 0.0001 | 0.0003 | 0.0003 | 0.48 | 0.1515 |
| mmu-miR-485-3p | 0.0036 | 0.0009 | 0.0016 | 0.0006 | 0.44 | **0.0319** |
| mmu-miR-488-3p | ND |  | ND |  |  |  |
| mmu-miR-489-3p | 0.0006 | 0.0003 | ND |  |  |  |
| mmu-miR-509-3p | ND |  | ND |  |  |  |
| mmu-miR-598-3p | 0.0027 | 0.0019 | 0.0010 | 0.0002 | 0.39 | 0.2134 |
| mmu-miR-652-3p | 0.0688 | 0.0277 | 0.0366 | 0.0044 | 0.53 | 0.1176 |
| mmu-miR-7a-5p | 0.0125 | 0.0008 | 0.0043 | 0.0023 | 0.35 | **0.0043** |
| mmu-miR-9-5p | 0.0004 | 0.0002 | ND |  |  |  |
| mmu-miR-9-3p | ND |  | ND |  |  |  |
| mmu-miR-92a-3p | 1.2146 | 0.6501 | 0.6451 | 0.0617 | 0.53 | 0.2055 |
| mmu-miR-93-5p | 0.6479 | 0.2671 | 0.3486 | 0.1299 | 0.54 | 0.1558 |
| mmu-miR-98-5p | 0.0061 | 0.0023 | 0.0034 | 0.0037 | 0.57 | 0.3524 |

**Sup. Table 4**

Comparison of plasmatic levels of evaluated miRNAs from 14-15 months-old 3xTg-AD and WT mice reveals AD-like pathology-related significant differences in the circulating miRNA profile between old mice. The table indicates the miRNA, the average relative abundance and the respective standard deviation (s) for each group, the fold-change and the p-value derived from the “t-test”. Relative abundance was through the difference of the threshold cycle (Ct) of each miRNA and the Ct of the miR-39 of *C. elegans* mimic (ΔCt) using the formula 2^-ΔCt^. Fold-changes between the 3xTg-AD and WT of 14-15 months groups were calculated using the formula 2^-ΔΔCt^.

| miRNA | WT of 14-15 months | | 3xTg-AD of 14-15 months | | Fold-change | p-value  “t-test” |
| --- | --- | --- | --- | --- | --- | --- |
|  | Relative abundance | s | Relative abundance | s |  |  |
| mmu-let-7b-5p | 0.2725 | 0.0343 | 0.3016 | 0.0648 | 1.11 | 0.5296 |
| mmu-let-7c-5p | 0.3838 | 0.0387 | 0.3344 | 0.0919 | 0.87 | 0.4391 |
| mmu-let-7d-5p | 0.0856 | 0.0243 | 0.0935 | 0.0403 | 1.09 | 0.7847 |
| mmu-let-7e-5p | 0.1476 | 0.0216 | 0.1461 | 0.0522 | 0.99 | 0.9654 |
| mmu-let-7i-5p | 0.1858 | 0.0575 | 0.1662 | 0.0540 | 0.89 | 0.6891 |
| mmu-miR-101a-3p | 0.0476 | 0.0246 | 0.0240 | 0.0076 | 0.51 | 0.1883 |
| mmu-miR-101b-3p | 0.0538 | 0.0301 | 0.0250 | 0.0064 | 0.46 | 0.1802 |
| mmu-miR-105 | ND |  | ND |  |  |  |
| mmu-miR-106b-5p | 0.3498 | 0.1147 | 0.3346 | 0.0884 | 0.96 | 0.8650 |
| mmu-miR-107-3p | 0.0027 | 0.0007 | 0.0026 | 0.0011 | 0.97 | 0.9141 |
| mmu-miR-124-3p | 0.0179 | 0.0156 | 0.0034 | 0.0020 | 0.19 | 0.1855 |
| mmu-miR-125b-5p | 0.2427 | 0.0549 | 0.1617 | 0.0199 | 0.67 | 0.0742 |
| mmu-miR-126-5p | 0.1754 | 0.0495 | 0.1408 | 0.0486 | 0.80 | 0.4359 |
| mmu-miR-128-3p | 0.0389 | 0.0080 | 0.0362 | 0.0059 | 0.93 | 0.6600 |
| mmu-miR-130a-3p | 0.0569 | 0.0060 | 0.0626 | 0.0257 | 1.10 | 0.7293 |
| mmu-miR-132-3p | 0.0160 | 0.0017 | 0.0077 | 0.0004 | 0.48 | **0.0013** |
| mmu-miR-133b-3p | 0.0230 | 0.0220 | 0.0178 | 0.0098 | 0.77 | 0.7261 |
| mmu-miR-134-5p | 0.0005 | 0.0002 | 0.0008 | 0.0003 | 1.59 | 0.2253 |
| mmu-miR-135b-5p | ND |  | ND |  |  |  |
| mmu-miR-138-5p | 0.0160 | 0.0048 | 0.0076 | 0.0004 | 0.47 | **0.0370** |
| mmu-miR-139-5p | 0.0904 | 0.0054 | 0.0616 | 0.0100 | 0.68 | **0.0117** |
| mmu-miR-140-5p | 0.0221 | 0.0048 | 0.0243 | 0.0082 | 1.10 | 0.7126 |
| mmu-miR-146a-5p | 0.2004 | 0.0101 | 0.0910 | 0.0188 | 0.45 | **0.0009** |
| mmu-miR-146b-5p | 0.0645 | 0.0080 | 0.0272 | 0.0085 | 0.42 | **0.0053** |
| mmu-miR-148b-3p | 0.0244 | 0.0116 | 0.0160 | 0.0048 | 0.65 | 0.3085 |
| mmu-miR-151-3p | 0.0142 | 0.0016 | 0.0122 | 0.0019 | 0.86 | 0.2370 |
| mmu-miR-152-3p | 0.0193 | 0.0077 | 0.0117 | 0.0040 | 0.61 | 0.2067 |
| mmu-miR-15a-5p | 0.0568 | 0.0304 | 0.0719 | 0.0263 | 1.27 | 0.5493 |
| mmu-miR-15b-5p | 0.1333 | 0.0428 | 0.1032 | 0.0390 | 0.77 | 0.4197 |
| mmu-miR-181a-5p | 0.0168 | 0.0041 | 0.0172 | 0.0031 | 1.03 | 0.8852 |
| mmu-miR-181a1-3p | 0.0005 | 0.0002 | 0.0005 | 0.0003 | 1.00 | 0.9900 |
| mmu-miR-181c-5p | 0.0152 | 0.0043 | 0.0162 | 0.0074 | 1.07 | 0.8468 |
| mmu-miR-181d-5p | 0.0200 | 0.0024 | 0.0194 | 0.0045 | 0.97 | 0.8468 |
| mmu-miR-191-5p | 0.2947 | 0.0483 | 0.2818 | 0.0393 | 0.96 | 0.7377 |
| mmu-miR-193b-3p | 0.0168 | 0.0023 | 0.0193 | 0.0026 | 1.15 | 0.2810 |
| mmu-miR-194-5p | 0.0892 | 0.0241 | 0.0740 | 0.0267 | 0.83 | 0.5062 |
| mmu-miR-195a-5p | 2.1263 | 0.2812 | 2.2807 | 0.6503 | 1.07 | 0.7251 |
| mmu-miR-19b-3p | 0.3347 | 0.1282 | 0.2562 | 0.0557 | 0.77 | 0.3856 |
| mmu-miR-203-3p | 0.1242 | 0.0413 | 0.0849 | 0.0410 | 0.68 | 0.3073 |
| mmu-miR-20a-5p | 0.4233 | 0.1385 | 0.3740 | 0.1224 | 0.88 | 0.6681 |
| mmu-miR-20b-5p | 0.1918 | 0.0474 | 0.1778 | 0.0717 | 0.93 | 0.7911 |
| mmu-miR-22-3p | 1.6335 | 0.1637 | 1.2351 | 0.1543 | 0.76 | **0.0374** |
| mmu-miR-24-3p | 1.4870 | 0.1634 | 1.0586 | 0.1150 | 0.71 | **0.0206** |
| mmu-miR-26b-5p | 0.1623 | 0.0680 | 0.1234 | 0.0246 | 0.76 | 0.4041 |
| mmu-miR-27a-3p | 1.3101 | 0.4461 | 0.6975 | 0.1204 | 0.53 | 0.0833 |
| mmu-miR-28c | 0.0020 | 0.0007 | 0.0018 | 0.0006 | 0.88 | 0.6738 |
| mmu-miR-298-5p | 0.0009 | 0.0002 | 0.0033 | 0.0023 | 3.76 | 0.1440 |
| mmu-miR-29a-3p | 1.0110 | 0.1881 | 0.6036 | 0.0613 | 0.60 | **0.0234** |
| mmu-miR-29b-3p | 0.0828 | 0.0269 | 0.0637 | 0.0078 | 0.77 | 0.3017 |
| mmu-miR-29c-3p | 0.9583 | 0.1753 | 0.5189 | 0.0609 | 0.54 | **0.0148** |
| mmu-miR-302a-5p | ND |  | ND |  |  |  |
| mmu-miR-302b-5p | ND |  | ND |  |  |  |
| mmu-miR-30a-5p | 0.5814 | 0.0252 | 0.5054 | 0.0540 | 0.87 | 0.0920 |
| mmu-miR-30d-5p | 0.2527 | 0.0338 | 0.2177 | 0.0321 | 0.86 | 0.2632 |
| mmu-miR-30e-5p | 0.5464 | 0.0544 | 0.4927 | 0.0518 | 0.90 | 0.2838 |
| mmu-miR-320-3p | 0.1075 | 0.0190 | 0.0936 | 0.0060 | 0.87 | 0.2912 |
| mmu-miR-328-3p | 0.0658 | 0.0188 | 0.0781 | 0.0081 | 1.19 | 0.3564 |
| mmu-miR-33-5p | 0.0082 | 0.0058 | 0.0062 | 0.0016 | 0.77 | 0.6134 |
| mmu-miR-337-3p | 0.0016 | 0.0007 | 0.0018 | 0.0008 | 1.14 | 0.7484 |
| mmu-miR-338-3p | 0.0073 | 0.0030 | 0.0056 | 0.0022 | 0.76 | 0.4692 |
| mmu-miR-339-5p | 0.0404 | 0.0082 | 0.0486 | 0.0091 | 1.20 | 0.3054 |
| mmu-miR-342-3p | 0.1719 | 0.0526 | 0.1109 | 0.0093 | 0.64 | 0.1188 |
| mmu-miR-346-5p | 0.0006 | 0.0002 | 0.0016 | 0.0006 | 2.98 | **0.0379** |
| mmu-miR-34a-5p | 0.0167 | 0.0048 | 0.0085 | 0.0024 | 0.51 | 0.0580 |
| mmu-miR-376b-3p | 0.0016 | 0.0007 | 0.0010 | 0.0001 | 0.67 | 0.2400 |
| mmu-miR-381-3p | 0.0006 | 0.0005 | 0.0007 | 0.0001 | 1.18 | 0.7456 |
| mmu-miR-409-3p | 0.0008 | 0.0003 | 0.0009 | 0.0002 | 1.14 | 0.6135 |
| mmu-miR-431-5p | 0.0011 | 0.0006 | 0.0013 | 0.0004 | 1.20 | 0.6164 |
| mmu-miR-433-3p | 0.0021 | 0.0007 | 0.0023 | 0.0003 | 1.13 | 0.5922 |
| mmu-miR-455-5p | 0.0014 | 0.0001 | 0.0012 | 0.0006 | 0.88 | 0.6637 |
| mmu-miR-484 | 0.2823 | 0.0651 | 0.2338 | 0.0204 | 0.83 | 0.2865 |
| mmu-miR-485-5p | 0.0002 | 0.0001 | 0.0003 | 0.0003 | 1.42 | 0.6410 |
| mmu-miR-485-3p | 0.0011 | 0.0001 | 0.0016 | 0.0006 | 1.52 | 0.1965 |
| mmu-miR-488-3p | ND |  | ND |  |  |  |
| mmu-miR-489-3p | ND |  | ND |  |  |  |
| mmu-miR-509-3p | ND |  | ND |  |  |  |
| mmu-miR-598-3p | 0.0013 | 0.0003 | 0.0010 | 0.0002 | 0.79 | 0.2993 |
| mmu-miR-652-3p | 0.0492 | 0.0088 | 0.0366 | 0.0044 | 0.74 | 0.0903 |
| mmu-miR-7a-5p | 0.0074 | 0.0013 | 0.0043 | 0.0023 | 0.58 | 0.1081 |
| mmu-miR-9-5p | ND |  | ND |  |  |  |
| mmu-miR-9-3p | ND |  | ND |  |  |  |
| mmu-miR-92a-3p | 0.6599 | 0.1835 | 0.6451 | 0.0617 | 0.98 | 0.9015 |
| mmu-miR-93-5p | 0.3036 | 0.0576 | 0.3486 | 0.1299 | 1.15 | 0.6123 |
| mmu-miR-98-5p | 0.0017 | 0.0010 | 0.0034 | 0.0037 | 2.05 | 0.4681 |
