# Supplementary material for: Identification of age- and disease-related alterations in circulating miRNAs in a mouse model of Alzheimer's disease
Source: Front Cell Neurosci. 2015 Feb 19;9:53. doi: 10.3389/fncel.2015.00053 (PMC4333818; doi:10.3389/fncel.2015.00053)
Supplement: Supplementary file 2 [file image1.pdf]

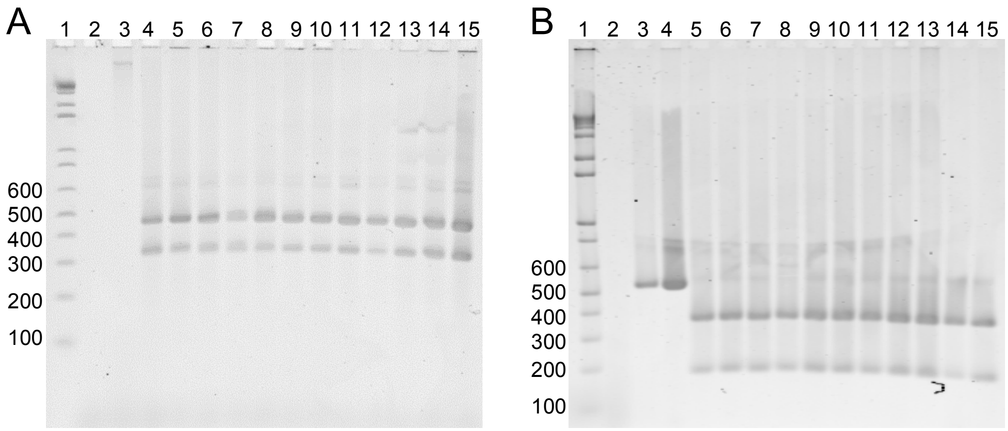

Sup. Fig. 1. Representative scans of agarose gels showing the amplification and digestion products of human transgenes harbored by the 3xTg-AD mice. (1) The molecular weight marker, (2) a negative control without DNA, and (3) a DNA of a WT mouse. (A) (4-15) APP_{Swe} and tau_{P301L} amplification products of 500 bp and 320 bp, respectively. (B) (4) An amplification product prior to digestion, and (5-15) digestion products of 350 bp and 180 bp corresponding to the presence of $PS1_{M146V}$ mutation in the 3xTg-AD mice.
